# Alternative polyadenylation produces multiple 3’ untranslated regions of odorant receptor mRNAs in mouse olfactory sensory neurons

**DOI:** 10.1186/s12864-019-5927-3

**Published:** 2019-07-12

**Authors:** Mohamed Doulazmi, Cyril Cros, Isabelle Dusart, Alain Trembleau, Caroline Dubacq

**Affiliations:** 1 CNRS, Institut de Biologie Paris Seine, Biological adaptation and ageing, B2A, Sorbonne Université, F-75005 Paris, France; 2 CNRS, INSERM, Institut de Biologie Paris Seine, Neuroscience Paris Seine, NPS, Sorbonne Université, F-75005 Paris, France; 3 Present Address: Columbia University, New York, NY 10027 USA

**Keywords:** Odorant receptors, Olfr genes, 3′ untranslated region, mRNA isoforms, Alternative polyadenylation, Adult olfactory mucosa, Olfactory sensory neuron, IsoSCM

## Abstract

**Background:**

Odorant receptor genes constitute the largest gene family in mammalian genomes and this family has been extensively studied in several species, but to date far less attention has been paid to the characterization of their mRNA 3′ untranslated regions (3’UTRs). Given the increasing importance of UTRs in the understanding of RNA metabolism, and the growing interest in alternative polyadenylation especially in the nervous system, we aimed at identifying the alternative isoforms of odorant receptor mRNAs generated through 3’UTR variation.

**Results:**

We implemented a dedicated pipeline using IsoSCM instead of Cufflinks to analyze RNA-Seq data from whole olfactory mucosa of adult mice and obtained an extensive description of the 3’UTR isoforms of odorant receptor mRNAs. To validate our bioinformatics approach, we exhaustively analyzed the 3’UTR isoforms produced from 2 pilot genes, using molecular approaches including northern blot and RNA ligation mediated polyadenylation test. Comparison between datasets further validated the pipeline and confirmed the alternative polyadenylation patterns of odorant receptors. Qualitative and quantitative analyses of the annotated 3′ regions demonstrate that 1) Odorant receptor 3’UTRs are longer than previously described in the literature; 2) More than 77% of odorant receptor mRNAs are subject to alternative polyadenylation, hence generating at least 2 detectable 3’UTR isoforms; 3) Splicing events in 3’UTRs are restricted to a limited subset of odorant receptor genes; and 4) Comparison between male and female data shows no sex-specific differences in odorant receptor 3’UTR isoforms.

**Conclusions:**

We demonstrated for the first time that odorant receptor genes are extensively subject to alternative polyadenylation. This ground-breaking change to the landscape of 3’UTR isoforms of Olfr mRNAs opens new avenues for investigating their respective functions, especially during the differentiation of olfactory sensory neurons.

**Electronic supplementary material:**

The online version of this article (10.1186/s12864-019-5927-3) contains supplementary material, which is available to authorized users.

## Background

Olfactory sensory neurons (OSNs) are the receptor cells of the mammalian olfactory system. OSN cell bodies are located in the olfactory mucosa (OM), a tissue covering the posterior/dorsal part of the nasal cavity. OSN axons project to the forebrain, into the olfactory bulb where they establish synaptic connections with dendrites of principal cells and interneurons within neuropils called glomeruli [1]. Odorant receptors (ORs), encoded by Olfr genes, are G Protein-Coupled Receptors (GPCRs) which were first identified as receptors of odorant molecules present at the surface of the OM [2]. Soon after the cloning of the first Olfr genes, it was shown that they constitute a very large family of genes [2–4], and that each OSN expresses only one Olfr gene from the full repertoire, in a monoallelic manner [5]. Such monogenic and monoallelic expression of Olfr genes in OSNs suggests tight genetic regulation. Furthermore, in terms of projections, it was shown that all OSNs expressing a given Olfr gene are scattered in large zones of the OM, but that their axons converge onto a small number of glomeruli [6–8]. Very interestingly, in addition to their role as chemoreceptors, ORs are involved in these two processes: ORs ensure their own monogenic and monoallelic expression [9], and play a critical role in guiding OSN axons towards their appropriate glomeruli [10–13]. Given the triple role played by Olfr genes in OSNs, and in view of deciphering the molecular and cellular mechanisms involved in these different functions, it is critical to fully characterize their structure and expression.

At the genomic and transcriptomic levels, the coding DNA sequence (CDS) and 5′ untranslated region (5’UTR) of Olfr genes have been extensively studied since the discovery of the gene family in the 1990s [2]. Olfr genes belong to the GPCR superfamily and most of them were first identified by CDS homology search. Phylogenetic analysis of Olfr subfamilies, genomic distribution in clusters, and polymorphism studies were done on CDSs [14–16]. Alternatively, 5’UTRs of Olfr genes were characterized through studies focusing on transcription start site identification and promoter analyses [17–21]. Early studies of Olfr cDNAs showed that, whereas introns are frequent (in low number) in 5’UTRs, they are very rare in Olfr CDSs [14, 15, 17]. Moreover, most Olfr expression studies were mainly based on CDS expression analyses [22–24]. In contrast, 3′ untranslated regions (3’UTRs) of Olfr mRNAs are as yet poorly documented in databases and rarely included in Olfr studies [25–27].

The lack of knowledge regarding the 3’UTRs of Olfr mRNAs is detrimental, given the well-established critical functions of 3’UTRs in regulating mRNA stability/degradation, translation repression/activation and subcellular localization [28]. Even more importantly, high throughput RNA sequencing during the past decade provided strong evidence for widespread alternative polyadenylation (APA) of mRNAs in eukaryotes, leading to the expression, for many genes, of several transcript isoforms with 3’UTRs of different lengths [29]. This APA turned out to be dynamically regulated during development [30], with lengthening or shortening of 3’UTR isoforms as an organism develops or a neuronal cell population differentiates [31–33]. At the subcellular level within developing neurons, APA is used to produce different mRNA isoforms having specific localization (i.e. axonal vs. cell body localization [34–38]). Furthermore, it was shown that APA is regulated in an activity dependent manner in adult neurons [39], where alternative 3’UTRs modify the localization, regulatory potential and plasticity of mRNAs in neuronal compartments [30].

To document the 3’UTRs of Olfr mRNAs in the mouse, and to determine to what extent these mRNAs are subject to APA, we developed a dedicated strategy based on computational analyses of previously published RNA-Seq datasets obtained from adult mouse whole olfactory mucosa [26, 40].

Given that Olfr genes constitute the largest gene family in mammalian genomes (including around ~ 1400 Olfr genes or pseudogenes in the mouse genome [3]), a high throughput approach was required. We had to take into account very specific properties of the Olfr family, in addition to the number and the similarity of the Olfr sequences that may challenge the specificity of the techniques. In particular, due to their monogenic and monoallelic expression [5], individual Olfr genes are expressed only in small subpopulations of OSNs and the resulting global low expression level of most of the Olfr genes in the OM may affect the annotation accuracy. Ibarra-Soria and colleagues [26] succeeded in generating 913 Olfr gene models from adult OM RNA-Seq data, thus bypassing the high similarity/low expression concerns. However, classical RNA-Seq annotation tools (e.g. Cufflinks) used by these authors and others [26, 27] are known to be poorly efficient in accurately identifying APA sites and the resulting 3’UTR isoforms [41]. In the present study, we re-analyzed Ibarra-Soria’s dataset with the Isoform Structural Change Model (IsoSCM) method, dedicated to 3’UTR annotation in ab initio assemblies [41], and compared with the Cufflinks method. The parameters for the reads alignment and annotation steps were set to optimize the accurate and comprehensive identification of the Olfr 3’UTR ends. In addition, we validated the annotations obtained for selected representative Olfr genes by independent experimental characterizations.

## Results

### Current documentation on Olfr 3’UTRs is not consistent, and likely underestimates their diversity

**Fig. 1:**
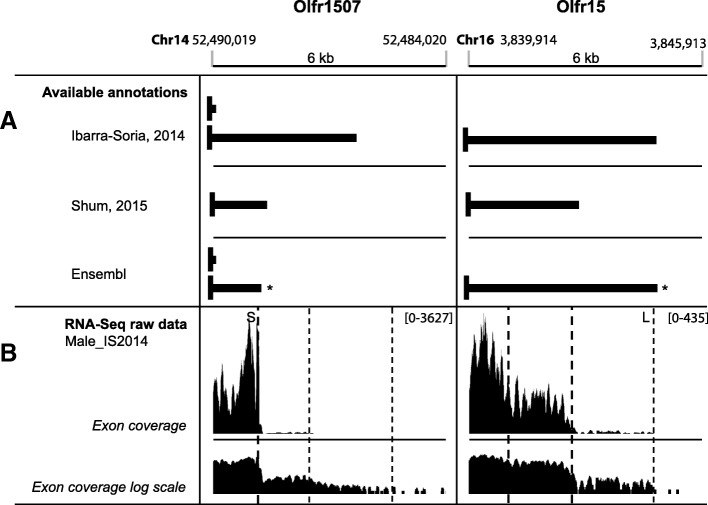
Comparison between available annotations for Olfr1507 and Olfr15 3’UTR isoforms and RNA-Seq raw data. **a** Upper panel. 3’UTRs from Ibarra-Soria et al. [26] RNA-Seq study (Dataset S6 in this paper). Center panel. 3’UTRs from Shum et al. [27] RNA-Seq study (File 013 Additional File 26 Table S3 in this paper). An alternative 3’UTR for Olfr15 due to 3′ length variation is mentioned (File 010 Additional File 3: Table S2 in [27]) but not described. Bottom panel. 3’UTRs in Ensembl release 89 [45].*: 3’UTRs deduced from RNA-Seq annotation (raw data in [26]) by the Havana project: ENSMUST00000206062.3 for Olfr1507; ENSMUST00000214590.1/ENSMUST00000214238.1 for Olfr15. Black vertical box: CDS end; black horizontal bar: 3’UTR. **b** Exon coverage, linear scale (top panel) and log scale (bottom panel), from olfactory mucosa RNA-Seq data of 3 adult male mice (male_IS2014, from [26]). Dashed lines indicate sustained drop of coverage that may be visually interpreted as 3′ ends; bold dashed lines indicate quantitatively major isoforms. Olfr1507 coverage suggests a short major 3’UTR isoform (S, previously described in **a**), and at least 2 additional minor 3’UTR isoforms with longer 3’UTRs (not described in **a**). Olfr15 coverage suggests 2 major 3’UTR isoforms (shorter than the 3’UTRs previously described in **a**), and a long minor 3’UTR isoform (L, previously described in **a**)

Until recently, most Olfr genes in databases showed no 3’UTR sequences. As an example, only 17 or 5 Olfr genes have one or multiple polyadenylation sites described in their annotated 3’UTR or extended 3′ region in 2 databases specific for 3’UTRs, PolyA_DB 3 [42] or APADB [43], respectively. Improved annotations of Olfr 3’UTR sequences were generated from male and female OM RNA-Seq data produced by Ibarra-Soria and colleagues [26]. In 2015, Shum and colleagues published a study focused on the analysis of Olfr 3’UTRs and established a description of the 3’UTRs for 554 Olfr genes from adult female whole OM RNA-Seq data [27]. At the end of 2016, gene models for mouse Olfr genes were updated by the Havana project based on Ibarra-Soria’s RNA-Seq data (Ensembl 87 release [44, 45]), and Olfr genes now have 3′ annotations in mouse genome databases. However, these 3′ annotations are not fully consistent with either the Shum or the Ibarra-Soria analyses. As an example, in the Ensembl database, Olfr1507 (also known as MOR28 or MOR244.1) shows 2 alternative 3’UTRs, one described by Ibarra-Soria and colleagues, and the other by Shum and colleagues (Fig. 1a). A third isoform identified by Ibarra-Soria et al., however, is excluded from the database. In the same Ensembl database, Olfr15 (also known as MOR256.17) shows a single 3’UTR, described by Ibarra-Soria et al., whereas Shum et al. demonstrated a shorter 3’UTR for this Olfr (Fig. 1a). In this latter work, an alternative 3’UTR was mentioned for Olfr15, but not described (Additional file 3: Table S2 in [27]).

In an attempt to clarify this issue, we went back to the raw data of Ibarra-Soria and colleagues [26], and noticed that there were discrepancies between the 3′ annotations and the RNA-Seq data. A visual inspection of the read coverage argues in favour of 2 major 3’UTR isoforms for Olfr15, shorter than the Ensembl annotation, which may represent a long isoform expressed at a low level (Fig. 1b). In the case of Olfr1507, the longer 3’UTR isoform annotated in Ensembl clearly appears as the major 3’UTR. While even longer additional 3’UTR isoform(s) of Olfr1507 most probably exist at a lower but detectable level, the shorter Ensembl isoform does not show up from the read coverage profile (Fig. 1b). Although we cannot exclude that some variations in 3’UTRs of Olfr mRNAs may come from sex (males and females for Ibarra-Soria et al. vs. females only for Shum et al.), breeding, or other technical differences between the 2 studies, these 2 examples clearly show that the current annotations of Olfr 3’UTR isoforms are not satisfactory.

In addition to the ambiguity described above, the number of alternative 3’UTRs clearly seems underestimated in both studies. This problem is likely due to the fact that both RNA-Seq analyses used Cufflinks for annotation, which in the literature [41] appears inefficient for the identification of alternative RNA ends. Thus, we decided to test dedicated tools and to set up a workflow specifically designed for the accurate characterization of the 3’UTRs of Olfr mRNAs. We applied this approach to available RNA-Seq datasets from first adult male OM, and then adult female OM.

### Implementation of a dedicated computational pipeline to document the 3’UTR repertoire of mouse Olfr mRNAs

**Fig. 2:**
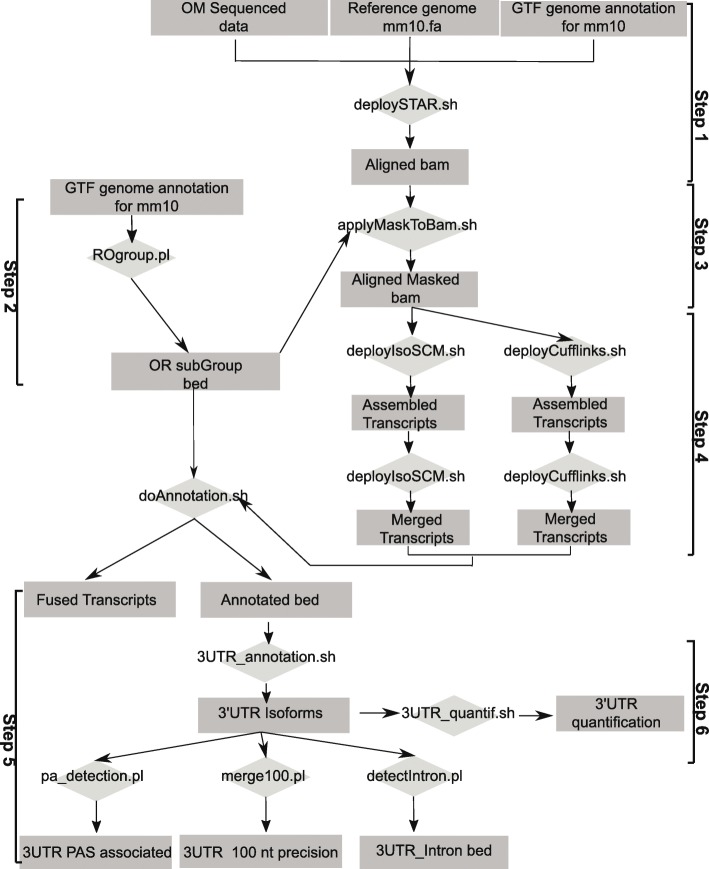
Computational pipeline for the characterization and analysis of Olfr 3’UTR isoforms from RNA-Seq datasets. The pipeline is divided into six steps: Step 1: RNA-Seq reads are mapped using STAR, and reference genome and annotation files. Step 2: A mask file in bed format, restricted to the Olfr loci in mouse, is generated. Step 3: The mask genome alignment output is obtained. Step 4: Full known and novel transcripts are reconstructed with IsoSCM or Cufflinks. Step 5: Reconstructed transcripts are characterized and analyzed in terms of annotation, 3’UTR isoforms identification, identification of predictive PASs at 3′ ends, merging (merge of 3’UTR isoforms from the same gene when 3′ ends are distant from less than 100 nt) and 3′ intron detection. Step 6: The relative abundances of the multiple 3’UTRs generated for the same Olfr gene are assessed by RNA-Seq quantification. Rectangles: input files or output files. Diamond shapes: bash shell and perl scripts.

Our workflow comprises 6 steps ultimately leading to the identification and relative quantification of all detectable Olfr 3’UTR isoforms (Fig. 2, see methods for a complete description). Briefly, we chose to base our computational pipeline on STAR software for alignment (Fig. 2, Step 1). Before the assembly step, we applied a mask restricted to genomic regions of Olfr genes, most of them being included in large genomic clusters [14, 15, 18, 46, 47] (Fig. 2, steps 2 and 3; Additional file 1: Table S1). In step 4, the IsoSCM algorithm was used for 3′ annotations. IsoSCM is an ab initio transcript assembly method implementing multiple change-point inference in terminal exon models [41]. IsoSCM distinguishes between local drops of coverage (due to biases) and sustained decrease of coverage (exon boundary change-point), and it avoids fragmentation due to small gaps in coverage. Among the available methods for 3’UTR annotation from RNA-Seq data, we chose IsoSCM for the following reasons: it allows more than two 3’UTRs per gene (as compared to PHMM method [48]), it is able to detect novel polyA sites independently of genome annotation (as compared to the DaPars [49, 50], 3USS [51], Roar [52], QAPA [53] or TAPAS [54] reference-based methods), it is based on change-point analysis (as compared to GETUTR [55], based on read coverage smoothing), it is not focused on samples comparison (as compared to ChangePoint [56]) and it is not dependent on 3’end sequencing (as compared to the IntMAP [57] or CSI-UTR [58] integrative methods). The optimization of key parameters of IsoSCM to reach a compromise between sensitivity and specificity are detailed in the methods section, and Additional file 2: Figure S1. In parallel, the data were processed in step 4 with the Cufflinks assembly method, to allow comparison with a previous study aiming at annotating the Olfr 3’UTRs [27]. In Step 5, transcripts were reconstructed. Furthermore, because 3’UTR ends depend on the presence of functional polyadenylation (polyA) signals (PAS) [59], we implemented in the workflow a search for canonical PAS in the vicinity of the newly identified 3’ends (Fig. 2, step 5). Finally, for each Olfr gene, we used the quantitative coverage data (counts) in the different segments defined by the alternative 3′ ends downstream of the stop codon, and their respective length, to estimate the relative abundance of each 3’UTR isoform among the entire mRNA population (Fig. 2, step 6).

Ibarra-Soria and colleagues provided high-quality data, with over 138 million paired-end fragments from 3 C57BL/6 J adult male OMs (referred to as male_IS2014) [26]. During the setup of the pipeline on these data, we considered that the precision of 3′ end annotation cannot exceed 100 nt for the following reasons: the read size is 75 nt, the cleavage sites at a single polyA site are observed in a 24-nt window [60], and a sequencing depth of 200 reads/kb is sufficient to reach a 100-nt resolution in IsoSCM transcript assembly with a 90% true positive rate [41]. In the data set produced by Ibarra-Soria and colleagues, only 59.4% of the Olfr genes exceed the sequencing depth of 200 counts/kb (in the CDS) for which a 100-nt resolution is achieved. Indeed, Olfr genes are expressed in a monogenic and monoallelic manner in OSNs and therefore most Olfr mRNAs show low abundance in whole olfactory mucosa extracts, limiting the precision of 3′ end identification for Olfr genes (see below). We considered further the 3′ ends which were matched with a canonical PAS (AAUAAA or AUUAAA) situated in a [− 100;+ 100] window. Overall, in our analysis, 67.4% of the annotated 3′ ends are matched with a canonical polyA signal. Thus, we definitely used a precision window of 100 nt.

From the 1181 Olfr genes investigated in adult male olfactory mucosa (male_IS2014 dataset), we were able to annotate 715 Olfr genes (61.4% of the 1165 Olfr genes expressed, i.e. with at least 1 read in the CDS) with the IsoSCM pipeline (Additional file 3: Table S2) and 908 Olfr genes with Cufflinks. For these annotated Olfr genes, our pipeline was able to detect 1547 3’UTRs with IsoSCM vs. 946 3’UTRs with Cufflinks. Furthermore, the mean number of 3′ ends per Olfr is 1.03 and 1.04 in the Cufflinks-based analysis by Shum and colleagues (File 010 Additional file 3: Table S2 in [27]) and by ourselves (male_IS2014 dataset), respectively, whereas it reaches 2.16 in our IsoSCM analysis of this same dataset. Before exploring further the results obtained with our pipeline, we used 2 pilot genes to validate our in silico data.

### Extensive characterization of the 3’UTR isoforms of 2 pilot Olfr genes

**Fig. 3:**
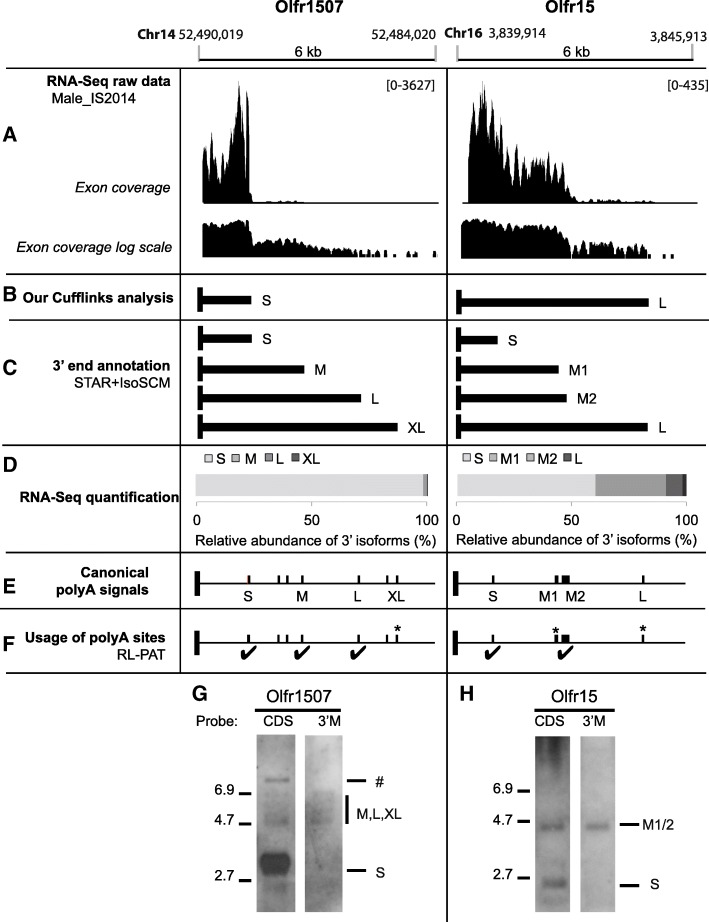
New annotations for Olfr1507 and Olfr15 3’UTR isoforms and experimental validation. **a** RNA-Seq raw data (see Fig. 1B for legend).** b** 3’UTRs generated using our own Cufflinks analysis. **c** 3’UTRs identified using our dedicated workflow applied to the male_IS2014 dataset with the STAR and IsoSCM algorithms. S: short, M: medium, L: long; XL: extra-long. Black vertical box: CDS end; black horizontal bar: 3’UTR. **d** Relative abundance of the alternative 3’UTR isoforms annotated in silico using our IsoSCM pipeline. **e** In silico validation of the 3’UTRs annotated using our IsoSCM pipeline by the presence of canonical PASs. Vertical bar = predicted canonical (AAUAAA or AUUAAA) PAS. **f** Experimental validation by RL-PAT (RNA Ligation mediated PolyAdenylation Test, Table 1). ✔ = genuine polyA site. *: XL site for Olfr1507, M1 and L sites for Olfr15 could not be tested by RL-PAT due to repeat sequences upstream of the polyA sites. **g** and **h** Experimental validation by northern blot. Total OM RNAs were separated on agarose/formaldehyde gels and transferred onto nitrocellulose membranes. The presence of Olfr mRNAs was detected following hybridization with DIG-labeled antisense probes either in the CDS region (CDS probe) or between the 3′S and 3’M ends (3’M probe; see Table 3 for detailed probe description). **g** The major isoform of the Olfr1507 mRNA shows a short 3’UTR (≈3-kb dark band with CDS probe, not detected with 3’M probe); additional isoforms having longer 3’UTRs are present at low abundance (4.7 to 6.9-kb faint bands with both probes); ♯ highest band (> 7 kb) corresponds to an intron-retaining transcript for Olfr1507 (Additional File 4: Fig. S2). **h** Olfr15 shows 2 major isoforms, the first one with a short 3’UTR (≈2.5-kb band with CDS probe, not detected with 3’M probe = 3′S), and the second one with a longer 3’UTR (≈4.5-kb band with both probes = 3’M1/2).

We undertook the extensive characterization of the 3’UTR isoforms produced by 2 pilot Olfr genes, Olfr1507 and Olfr15, belonging to distinct Class II odorant receptors subfamilies. In our hands, as for the two previous studies [26, 27], the Cufflinks’ method appears inefficient for the identification of alternative RNA ends (Fig. 3b). The annotated 3’UTRs obtained using our IsoSCM pipeline appear much more consistent, and even more precise than what could be presumed from a close visual inspection of RNA-Seq data for these 2 genes (Fig. 3a and c). For both Olfr1507 and Olfr15, 4 alternative 3’UTRs are identified. Some of these isoforms correspond to the 3’UTRs obtained with Cufflinks (Olfr1507 3′S; Olfr15 3’M and 3’L; Figs. 1a and 3b-c). Interestingly, some isoforms are newly-identified 3’UTRs: Olfr1507 3’M, 3’L and 3’XL; Olfr15 3′S. The shortest Olfr1507 3’UTR described in Ensembl was not reconstructed from our pipeline (see further analysis below).

Importantly, our pipeline includes a step for relative quantification of the different 3’UTR isoforms produced by a given Olfr gene. Olfr1507 3′S represents 98.1% of the Olfr1507 mRNAs. Olfr15 3′S represents only 60.3%, while Olfr15 3’M1 and 3’M2 together constitute 38.0% of the Olfr15 mRNAs (Fig. 3d). Remarkably, all the 3′ alternative isoforms identified for the 2 genes are matching with canonical PASs (Fig. 3e and Table 1).

**Table 1:** Alternative 3′ ends for Olfr1507 and Olfr15 revealed in silico, and their subsequent experimental validation

		RNA-Seq		RL-PAT			
Gene	PolyA site	3’end position (nt after stop)	Matching canonical PAS (nt after stop)^b^	Cleavage position (nt after stop)	Nb of clones sequenced	PolyA tail length (nt, mean ± SEM)	Matching canonical PAS (nt after stop)^e^
Olfr1507	XS	Not detected	Not relevant	92	2	19 ± 8	None
				114	2	17 ± 3	
	S	1193–1273^a^	AAUAAA (1263, 1281)	1283	7	44 ± 18	AAUAAA (1263, 1281)
				1290	1	11	
				1294	1	39	
	M	2593	AUUAAA (2628)	2653	1	86	AUUAAA (2628)
	L	4013	AAUAAA (4076)	4093	1	16	AAUAAA (4076)
				4109	2	16 ± 1	
				4112	1	46	
	XL	4943	AAUAAA (5049)^c^	No RL-PAT confirmation^d^			Not relevant
Olfr15	S	902	AAUAAA (901)	924	2	87 ± 1	AAUAAA (901)
				931	1	26	
				936	1	19	
	M1	2462	AUUAAA (2487), AAUAAA (2529)	No RL-PAT confirmation^d^			Not relevant
	M2	2742	AAUAAA (2665, 2675, 2685, 2806, 2798, 2806, 2814, 2822), AUUAAA (2790)	2816	1	22	AAUAAA (2798, 2806, 2814, 2822), AUUAAA (2790)
				2820	1	28	
				2824	1	31	
				2836	1	48	
	L	4762-4837^a^	AUUAAA (4717)	No RL-PAT confirmation^d^			Not relevant

^a^The 3’ ends distant of less than 100 nt were merged as a single polyA site. ^b^For each putative polyA site, predicted canonical PAS matching in a [− 100;+ 100] window from the RNA-Seq-deduced 3′ end are indicated. ^c^Note that for Olfr1507 3’XL isoform, the closest canonical PAS is slightly outside the 100-nt precision window. The cleavage site and polyA tail length were experimentally mapped using RL-PAT on adult male OM, further confirming the polyA site usage for most of the 3’UTR isoforms. ^d^XL site of Olfr1507, M1 and L sites of Olfr15 could not be confirmed by RL-PAT because no specific forward primer could be designed due to repeat sequences upstream of these polyA sites. ^e^For each confirmed polyA site, predicted canonical PAS matching in a [− 100;0] window from the identified cleavage sites are indicated

To confirm the biological existence of these 3’UTRs, we performed experimental characterization of the 8 putative polyA sites annotated for Olfr1507 and Olfr15.

First, we investigated the precise cleavage sites by RNA Ligation mediated PolyAdenylation Test (RL-PAT), an RT-PCR-based strategy. We confirmed the use of 5 out of the 8 polyA sites using this technique (Fig. 3f; Table 1). Unfortunately, the 3 last sites could not be validated using this method, due to technical limitations: the presence of upstream repeat sequences made it impossible to design specific primers for testing Olfr1507 3’XL, Olfr15 3’L and 3’M1.

Next, we demonstrated by northern blot the existence of alternative 3’UTR isoforms for both pilot genes using probes designed for the sequences either upstream (CDS probe) or downstream (3’M probe) of the proximal 3′ ends (Fig. 3g-h). The Olfr1507 3′S isoform appears as the major 3’UTR: whereas a very strong signal attributed to 3′S isoform is detected with the CDS probe, another probe designed downstream of the 3′S end reveals only faint bands corresponding to much less abundant longer 3’UTRs (3’M, 3’L or 3’XL) (Fig. 3g). A second band specifically detected with the CDS probe corresponds to a 5′ intron-retaining transcript displaying the S 3’UTR (Additional file 4: Figure S2). For Olfr15, 3′S and 3’M1/2 appear as two similarly dominant 3’UTR isoforms. Indeed, the Olfr15 3’M1 resides in a stretch of canonical PAS (11 AAUAAA or AUUAAA sequences from 2487 to 2822 after the stop codon) together with Olfr15 3’M2 (Fig. 3h). The 3’L isoform of Olfr15, not detected using northern blot, is most probably expressed at a very low level (below the sensitivity of this technique; Fig. 3h). The existence of an XS 3’UTR for Olfr1507 (described by Ibarra-Soria’s study and in the Ensembl database; Fig. 1a) was confirmed by RL-PAT, and this isoform is probably generated using a non-canonical PAS (Table 1). However, this 3’XS isoform is probably expressed at a very low level (below northern blot sensitivity – Fig. 3g). Very importantly, northern blot confirmed the relative abundances of the alternative 3’UTR isoforms determined using RNA-Seq quantification (Fig. 3d).

In conclusion, our experimental investigations performed on these two pilot genes confirmed the data obtained in silico using our IsoSCM pipeline, from both the qualitative (repertoire of 3’UTRs) and quantitative (relative abundances of alternative isoforms) points of view. Altogether, this analysis fully supports the expression of multiple 3’UTR isoforms produced by alternative polyadenylation, and it further shows that different Olfr genes may produce different patterns of Olfr mRNA isoforms in terms of relative abundance (i.e. 1 highly expressed isoform plus several others expressed at a low level for Olfr1507 vs. 2 isoforms expressed at similarly high level plus several others expressed at low level for Olfr15).

### Characterization of the full repertoire of detectable Olfr 3’UTRs using our IsoSCM pipeline

**Fig. 4:**
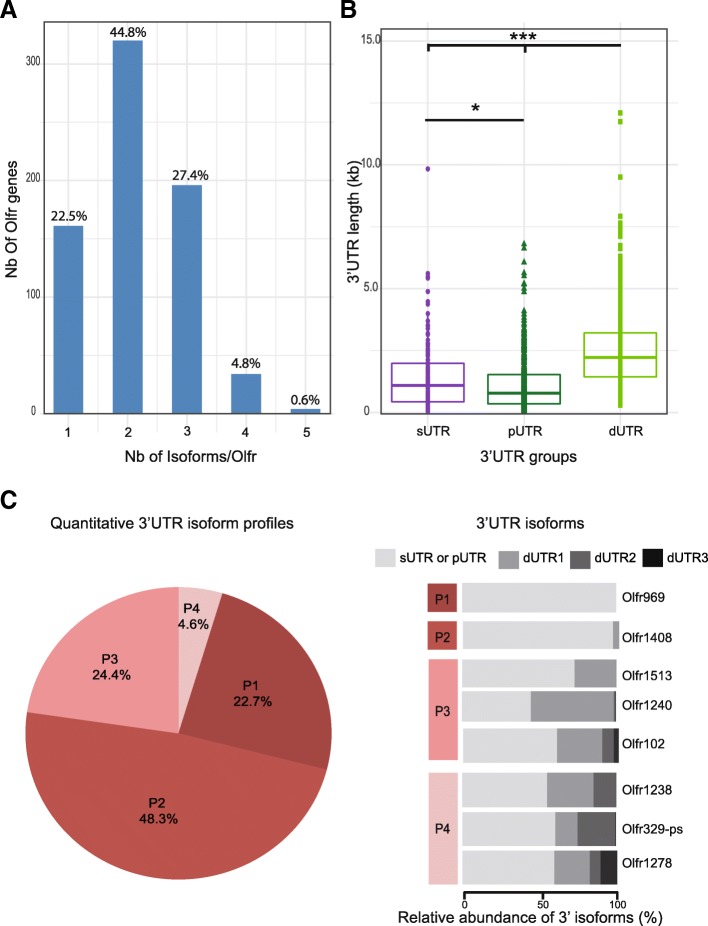
A large majority of Olfr mRNAs shows multiple 3’UTR isoforms produced through alternative polyadenylation. The whole male_IS2104 dataset was analyzed using our IsoSCM pipeline. **a** Distribution of numbers of 3’UTR isoforms per Olfr. **b** Distribution of length for 3’UTR groups; sUTR = single 3’UTR for Olfr genes without APA (purple); pUTR = proximal 3’UTR isoform (dark green) and dUTR = distal 3’UTR isoform(s) (light green) for Olfr genes with APA. One-way Kruskal-Wallis test (Chi square = 430.62, *p* < 0.0001, df = 2), followed by Nemenyi test (* *p* < 0.05, *** *p* < 0.001). **c** Distribution of Olfr genes in 4 distinct quantitative 3’UTR isoform profiles. P1 = Olfr genes without APA (their mRNAs present a single 3’UTR), P2 = Olfr genes with APA, the proximal 3’UTR isoform representing more than 80% of the mRNAs (typically, the proximal 3’UTR is the major isoform); P3 = Olfr genes with APA, the proximal 3’UTR isoform representing less than 80% and the sum of the most distal isoforms (dUTR2 to dUTR4) less than 10% (typically, the pUTR and dUTR1 are the main isoforms); P4 = Olfr genes with APA, the proximal 3’UTR isoform representing less than 80% and the sum of the most distal isoforms (dUTR2 to dUTR4) more than 10% (typically, these Olfr genes have 3 or more isoforms of quantitative importance). Typical examples for these 4 quantitative profiles are shown in the right panel

As mentioned above, our IsoSCM pipeline allowed the characterization of 1547 3’UTR isoforms, generated from the 715 Olfr genes annotated from the male_IS2014 dataset. As for our 2 pilot genes, the great majority of the annotated Olfr genes (77.5%) show multiple 3’UTRs due to alternative polyadenylation, 44.8% with 2 isoforms, 27.4% with 3 isoforms and 5.3% with 4 or 5 isoforms (Fig. 4a).

To determine if our pipeline could introduce a bias excluding rare alternative isoforms for Olfr genes expressed at low level, we analyzed the correlation between expression level (based on reads quantification in the CDS) and annotation of multiple isoforms. It was important to determine if Olfr genes for which we detected a single 3’UTR isoform were expressed at lower abundance as compared to Olfr genes for which we detected multiple 3’UTR isoforms. This statistical analysis shows that our pipeline can discriminate between single 3’UTRs and up to 3 multiple isoforms due to APA, independently of the expression level of the Olfr genes (Additional file 5: Figure S3A). Nevertheless, we probably underestimate the number of alternative isoforms for genes expressed at low level, as well as mRNA isoforms expressed at very low levels.

The median length of the 3’UTR isoforms for Olfr mRNAs, deduced in silico with our IsoSCM pipeline*,* is 1576 nt. This value is however variable from one Olfr 3’UTR to another, with two of them being longer than 10 kb (Fig. 4b). The median length of Olfr 3’UTRs in our IsoSCM analysis appears to be twice the 773 nt median length previously described by Shum and colleagues [27], and it is higher than the 1278 nt median length obtained with our Cufflinks pipeline on the same data (Mann Whitney, *p* < 0.0001). Considering that, among the 3’UTR isoforms characterized by our IsoSCM pipeline, some are the only one detected for a given Olfr, while others belong to a group of multiple isoforms generated through APA from a given Olfr gene, we distinguished 3 categories of 3’UTRs. The first category corresponds to the single 3’UTRs (only 1 isoform produced by the Olfr gene; sUTR). We further distinguished 2 additional categories for the multiple isoforms (as defined in [53]): the isoforms generated by the proximal APA site (pUTRs), and the ones generated by the other(s), thereafter called the distal UTR(s) (dUTRs). Depending on their number, dUTRs will be thereafter called dUTR1, dUTR2 etc., with the incremental number ordered according to their length. Among those 3 groups of 3’UTRs, both sUTRs (median length = 1088 nt) and dUTRs (median length = 2217 nt) annotated with the IsoSCM pipeline appear to be longer than the Olfr 3’UTRs previously described by others [27] (*p* < 0.001), whereas pUTRs (median length = 782 nt) reach similar length (*p* = 0.773). Strikingly, pUTRs are significantly shorter than sUTRs (One-way Kruskal-Wallis test, followed by Nemenyi test, *p* < 0.01; Fig. 4b). As expected, dUTRs are significantly longer than both pUTR and sUTRs (One-way Kruskal-Wallis test, followed by Nemenyi test, *p* < 0.001; Fig. 4b). The resultant median of length ratio dUTR1/pUTR for Olfr genes with multiple 3’isofoms reaches 2.58. Thus, our analysis using our IsoSCM pipeline allowed us to uncover a previously unsuspected much higher global length of the annotated Olfr 3’UTRs produced by APA, and demonstrated that alternative polyadenylation of Olfr mRNAs generates a global lengthening of 150% of their 3’UTRs.

Further hierarchical clustering with Principal Component Analysis (PCA) analysis (see methods) of the in silico relative quantification data led us to consider 4 distinct relative-abundance profiles of 3’UTRs (Fig. 4c). Results from PCA based on the relative abundance and length of alternative isoforms demonstrated that three principal components (PCs) had eigenvalues more than 1 (Kaiser’s criteria) and were retained. Cumulative variance explained by these three PCs was 86.51%. The P1 relative-abundance profile (22.7%) contains all Olfr genes producing a single 3’UTR (no APA). The P2 profile (48.3%) contains Olfr genes producing at least 2 isoforms, including a major proximal isoform, the sum of the distal isoforms representing less than 20% of the mRNAs. For the remaining Olfr genes producing at least 2 isoforms including one representing less than 80%, we distinguished two additional classes: a P3 profile (24.4%) for Olfr genes presenting a proximal and one distal isoform being of quantitative importance (the sum of the other distal isoforms representing less than 10% of all isoforms), and a P4 profile (4.6%) for Olfr genes producing 3 or more isoforms of quantitative importance (the sum of dUTR2 to dUTR4 isoforms representing more than 10% of the mRNAs). Thus 29% of annotated Olfr genes produce several 3’UTR isoforms of quantitative importance (P3 or P4 profiles), as for Olfr15 (Figs. 4c and 3d and h). In 71% of annotated Olfr genes an isoform represent more than 80% of the isoform either because it is a unique (P1 profile) or a major (P2 profile) isoform, as for the Olfr1507 pilot gene (Figs. 4c and 3d and g).

**Fig. 5:**
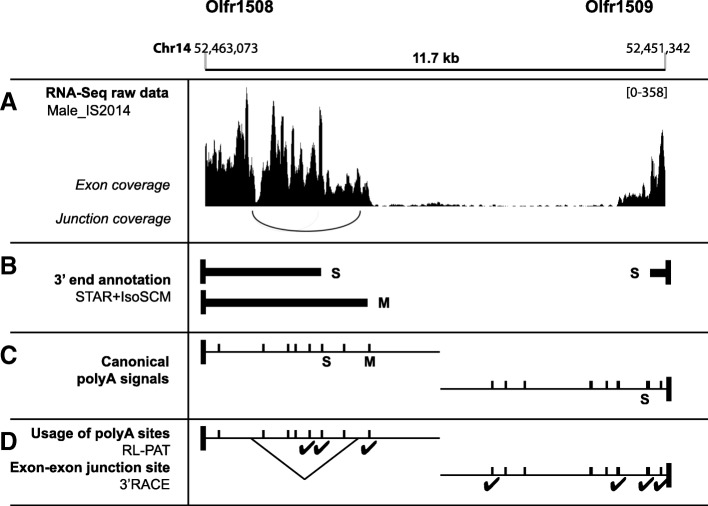
New annotations for Olfr1508 and Olfr1509 3’UTR isoforms and experimental validation. **a** RNA-Seq raw data within a 11.7-kb region comprising both Olfr1508 and Olf1509 3’UTRs (see Fig. 1b for legend). Junction coverage is shown for the Olfr1508 strand. **b** 3’UTRs identified using our IsoSCM pipeline processing the male_IS2014 dataset. Black vertical box: CDS end; black horizontal bar: 3’UTR. **c** In silico validation of the 3’UTRs annotated using our IsoSCM pipeline by the presence of canonical PASs. Vertical bar = predicted canonical (AAUAAA or AUUAAA) PAS. **d** Experimental validation by RL-PAT. ✔ = genuine polyA site. The optional splicing in the 3’M of Olfr1508 has been confirmed by 3’RACE (3′ Rapid Amplification of cDNA ends). S: short, M: medium

To characterize the contribution of alternative splicing to the 3′ alternative isoforms of Olfr mRNAs, we identified the introns included in this new 3’UTR repertoire. It turned out that only 13 Olfr genes display an obligatory or optional intron in their 3’UTRs with a depth > 1 (male_IS2014 dataset, Additional file 6: Table S3). As an example, the 3’M isoform of Olfr1508 is subject to an optional intron excision detected by STAR (Fig. 5a-c). This optional splicing was further confirmed by RT-PCR amplification (3’RACE, Rapid Amplification of cDNA Ends; Fig. 5d).

Overall, although a large majority of Olfr mRNAs shows alternative 3’UTR isoforms, mostly produced by APA, we observed great diversity in terms of length and/or relative abundance of these multiple isoforms. Moreover, a subset of Olfr mRNAs showed no detectable alternative 3’UTR using our pipeline, suggesting possible diverse regulation of APA among the Olfr gene family.

**Fig. 6:**
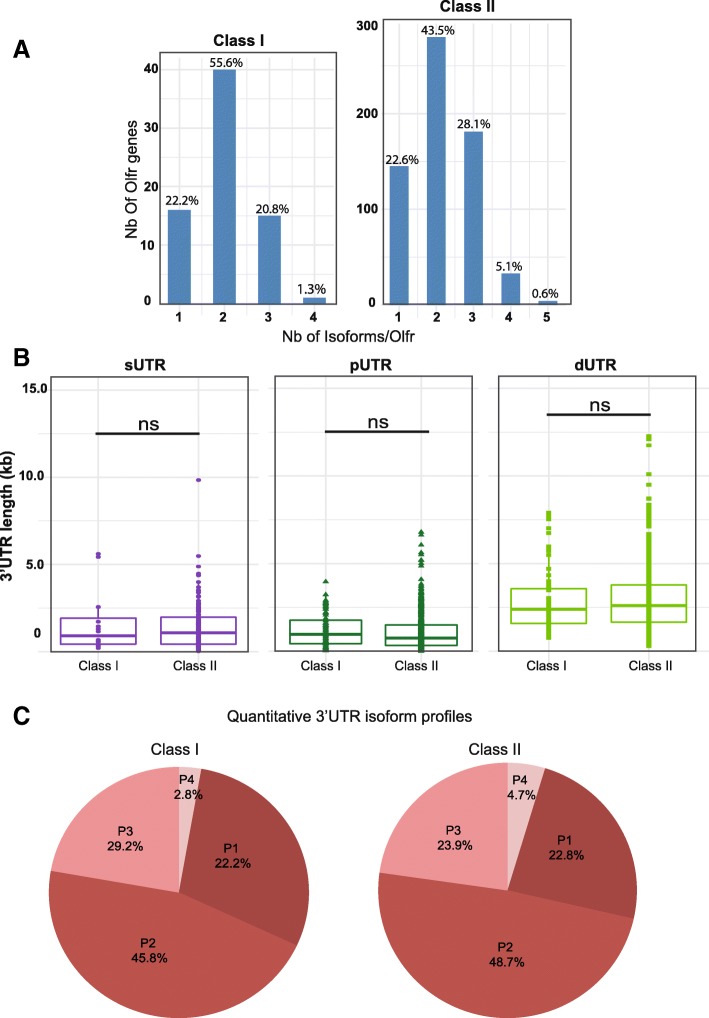
Class I and Class II Olfr mRNAs show similar patterns of alternative polyadenylation. **a** Distribution of numbers of 3’UTR isoforms per Olfr. **b** Comparison between Class I and Class II Olfr genes’ distribution of length for 3’UTR groups. Mann–Whitney test (ns *p* > 0.05). See Fig. 4 for the definition of the groups. **c** Distribution of Olfr genes in 4 distinct quantitative 3’UTR isoform profiles. See Fig. 4 for the definition of the profiles. A Fisher-Freeman-Halton exact test shows no significant difference (*p* = 0.76)

Among the Olfr gene family, the Class I Olfr genes represent a small sub-family (135 genes on chromosome 7) [14], and we wondered if APA was differently regulated for these Class I Olfr genes. Class I Olfr mRNAs are clearly less abundant than Class II Olfr mRNAs in the OM. However, we detected no bias associated with the expression level in the detection of one or multiple 3’UTR isoforms for the Class I Olfr genes (Additional file 7: Figure S4A). We discriminate between single 3’UTRs and up to 3 multiple isoforms due to APA independently of the expression level for Class II Olfr genes (Additional file 7: Figure S4B). We found 145 3’UTR isoforms produced from 72 Class I Olfr genes and 1400 3’UTR isoforms produced from 643 Class II Olfr genes. Both Class I and Class II Olfr genes display the massive APA demonstrated above (77.8 and 77.4% APA respectively; Fig. 6a). The median lengths of the sUTRs, pUTRs or dUTRs are not significantly different between Class I and Class II Olfr genes (Fig. 6b; Wilcoxon rank sum test: sUTR (*p* = 0.93), pUTR (*p* = 0.39), dUTR (*p* = 0.48)). Likewise, the quantitative profiles of the 3’UTR isoforms do not show any difference between Class I and Class II Olfr genes (Fig. 6c). Overall, these two Olfr gene sub-families display similar APA profiles (*p* = 0.7).

### Comparison of 3’UTR isoforms annotated from 4 datasets reveals robust 3’UTR isoforms in both sexes, and no sex-specific 3’UTRs

All the above analyses were done on the RNA-Seq dataset from 3 adult male OM published by Ibarra-Soria and colleagues in 2014 (male_IS2014) [26]. In the same paper, a second dataset was obtained from 3 adult female OM (referred to as female_IS2014). An independent experiment with both adult male (male_IS2017) and adult female (female_IS2017) datasets was recently published by the same authors [40]. We took advantage of the availability of these 4 datasets produced by the same laboratory to evaluate the robustness of our IsoSCM pipeline and to compare the Olfr 3’UTR repertoires between females and males.

#### Global comparative analysis of the annotations from the 4 datasets

We processed these data with our IsoSCM pipeline to annotate the Olfr 3’UTRs. While the absolute numbers of annotated Olfr genes and 3’UTR isoforms obtained in the 4 datasets differ (715, 834, 625 or 743 annotated Olfr genes and 1547, 1810, 1344 or 1565 3’UTR isoforms for the male_IS2014, male_IS2017, female_IS2014 or female_IS2017 datasets, respectively), these repertoires display a large overlap from one sample to another (Additional file 8: Figure S5A-D). As was the case for the male_IS2014 dataset, the detection of multiple (up to 3) 3’UTR isoforms did not depend on the expression level of the Olfr genes in the 3 other datasets (Additional file 5: Figure S3).

We however observed a differential expression of the Olfr genes between the samples from the 2 independent experiments (Additional file 8: Figure S5E-H). Whereas male and female samples from the same experiment had similar expression levels, samples from the same sex from different experiments show significantly different expression levels, with an increased normalized expression in the 2017 experiment as compared to the 2014 experiment. This was not surprising because these 2 experiments are not biological replicates; they differ in animal housing conditions, sample preparation (stranded vs. not), read length (100 vs. 75 nt) and sequencing technology (Illumina HiSeq 2500 vs. 2000). This may explain the increased number of Olfr genes with annotated 3’UTRs in the second experiment.

In addition, because the uncertainty regarding the position of polyadenylation sites reaches 200 nt between 2 annotations, we considered 3’UTR isoforms as “conserved” between 2 datasets when the length difference between the corresponding 3’UTRs annotated in the 2 datasets is less than 200 nt. According to this rule, 483 s/pUTRs (of 639 Olfr genes annotated in both male datasets) and 247 dUTR1s (of 561 Olfr genes with an annotated dUTR1 in at least one male dataset, and 446 in both male datasets) are conserved between the 2 male experiments (Additional file 3: Table S2). Since the coverage decreases along the 3′ regions, the most distal 3’UTRs (dUTR2, dUTR3 and dUTR4) show higher probability to be either missed or misplaced (due to reduced precision of the assembly). Accordingly, the length of same-rank UTRs is better conserved between the 2 male datasets for single or proximal 3’UTR isoforms (75.6% “conserved” s/pUTRs), than for the distal 3’UTR isoforms (55.4% “conserved” dUTR1s; 51.4% dUTR2s, 35.7% dUTR3s). The rare Olfr genes showing dUTR4 isoforms were not the same in the 2 male datasets. Therefore, we focused on sUTR, pUTR and dUTR1 3’UTR isoforms when comparing 3’UTR isoforms produced by individual Olfr genes in the different datasets.

**Fig. 7:**
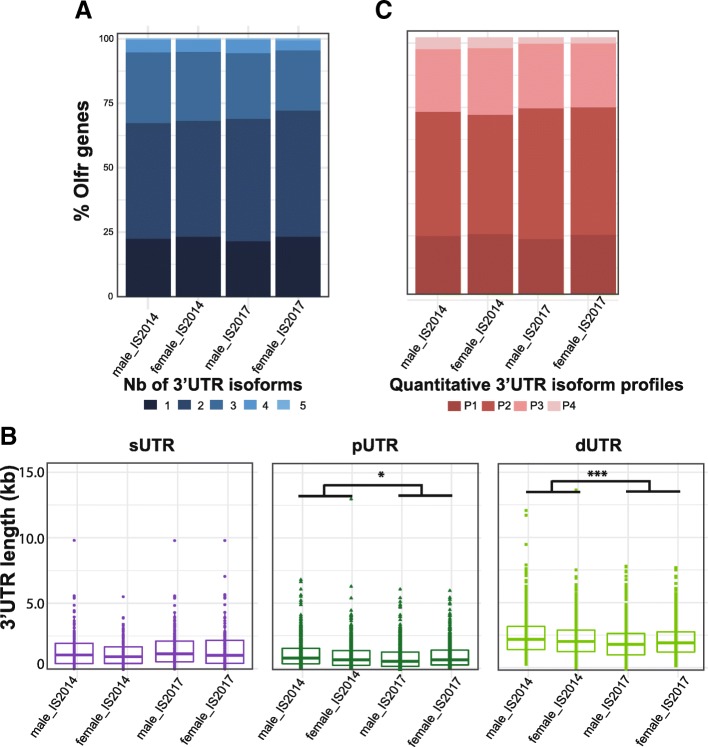
Comparison of the Olfr 3’UTR annotations between 4 datasets from adult olfactory mucosa. The male_IS2104, female_IS2014, male_IS2017 and female_IS2017 datasets were analyzed using our IsoSCM pipeline. **a** Distribution of numbers of 3’UTR isoforms per Olfr. A Fisher-Freeman-Halton exact test shows no significant difference (*p* = 0.75). **b** Distribution of sUTR, pUTR and dUTR lengths. The length of sUTRs (left panel) does not appear statistically different between the 4 datasets (two-way ANOVA: experiment effect, F_(*1, 656*)_ = 2.45, *p* > 0.05; sex effect, F_(*1, 656*)_ = 0.88, *p* > 0.05; no interaction). Whereas the length of pUTRs (middle panel) is significantly lower in the 2017 experiment as compared to the 2014 one (two-way ANOVA: experiment effect, F_(1, 2253)_ = 5.34, *p* = 0.03; sex effect, F_(1, 2253)_ = 0.18, *p* > 0.05; no interaction), this difference falls into the 200-nt uncertainty window and may thus not reflect a genuine difference between these 2 experiments. The length of dUTRs (right panel) is statistically different between the 4 datasets (two-way ANOVA: experiment effect, F_(*1, 3319*)_ = 17.38, *p* < 0.001; sex effect, F_(*1, 3319*)_ = 3.06, *p* > 0.05; no interaction). **c** Distribution of Olfr genes in 4 distinct quantitative 3’UTR isoform profiles. See Fig. 4 for the definition of the profiles. A Fisher-Freeman-Halton exact test shows no significant difference (*p* = 0.37)

The global analysis of these 4 datasets confirmed a substantial APA for Olfr mRNAs both in male and female (77.5, 78.4, 76.6 and 76.7% of the annotated Olfr genes in the male_IS2014, male_IS2017, female_IS2014 or female_IS2017 datasets; Fig. 7a). As for the first dataset of male_IS2014, we observed a large diversity in the length of the 3’UTRs, with significantly different lengths between the sUTRs, pUTRs and dUTRs within each dataset. There was no difference between the 4 datasets in terms of 3’UTR length for either sUTRs or pUTRs (Fig. 7b), or in their relative abundance profiles (Fig. 7c). However, a significant difference in dUTR length was observed between the 2 experiments (2014 vs. 2017), although not between male and female datasets (Fig. 7b). For all parameters examined between the 4 datasets, these global investigations thus did not reveal striking differences, except a length difference for dUTRs between the 2014 and 2017 experiments, probably due to the technical differences in these 2 experiments.

#### Comparative analysis of the annotations for individual Olfr genes common to the 4 datasets

Five hundred and one Olfr genes with at least one annotated 3’UTR isoform were retrieved in all 4 datasets. Our 2 pilot genes, Olfr1507 and Olfr15, turned out to belong to this group of 501 common Olfr genes. Their detailed in silico analysis globally confirmed the robustness of our pipeline across independent datasets, in terms of alternative 3′ ends and length of the 3’UTRs, even though minor differences were observed (Additional file 9: Figure S6; Additional file 10: Table S4). The absence of detection of 2 distinct 3′ ends for the 3’M1/2 PAS of Olfr15 could be attributed to the presence of a stretch of canonical PAS (see above). A very rare 3’XL was detected only in female_IS2014 but in neither male nor in female_IS2017 datasets. This discrepancy might be explained by the limit in sensitivity of our technique for isoforms expressed at a very low abundance. From a quantitative point of view, the relative abundance profiles of 3’UTR isoforms are not statistically different for these 2 genes across the 4 datasets (Fisher exact test Olfr1507 *p* = 1; Fisher exact test Olfr15 *p* = 0.24; Additional file 9: Figure S6).

This robustness encouraged us to further compare the annotations of Olfr genes between the 4 datasets (Additional file 3: Table S2). We first searched for isoforms that are experiment-specific i.e. not conserved in same-sex comparisons (male_IS2014 vs. male_IS2017 and female_IS2014 vs. female_IS2017) but conserved between males and females in both experimental series (male_IS2014 vs. female_IS2014 and male_IS2017 vs. female_IS2017 comparisons). Among the 501 common Olfr genes across the 4 datasets, we identified 27 experiment-specific s/pUTRs. Focusing on the 324 Olfr genes with multiple 3’UTR isoforms in the 4 datasets, we found 21 experiment-specific dUTR1s. On the other hand, we identified putative sex-specific isoforms, i.e. conserved in same-sex comparison but not conserved between males and females in both experimental series. Only 4 s/pUTRs (for Olfr133, Olfr1107, Olfr618 and Olfr1226) and 5 dUTR1s (for Olfr476, Olfr796, Olfr1226, Olfr653 and Olfr11271) display such an apparent sex difference (Additional file 3: Table S2). Thus, as far as we can conclude from this study of 501 Olfr genes, we identified only marginal possibilities of sex-associated APA events in the 3’UTRs of Olfr mRNAs, whereas some experiment-specific annotations of Olfr 3’UTRs may arise because of technical differences between the 2 sets of experiments. By contrast, we annotated 310 s/pUTRs and 114 dUTR1s that are fully conserved in the 4 datasets analyzed (independently of the experiment series or the sex of the mice, Additional file 3: Table S2). These annotations constitute a list of very robust 3’UTR isoforms for Olfr mRNAs. Finally, we found only 6 Olfr genes with introns in 3′ regions in the 4 datasets (Olfr1508, Olfr273, Olfr303, Olfr399, Olfr620, Olfr706), and no sex-specific splicing events (Additional file 6: Table S3).

Altogether, our data demonstrate that the new Olfr 3’UTR repertoire identified through our IsoSCM pipeline correspond to cognate 3’UTR ends expressed in the adult olfactory mucosa, and that the large majority of these in silico characterized 3’UTRs are robustly identified independently of the biological or technical variations introduced by the choice of the RNA-Seq datasets. Overall, it is likely that the few minor differences observed between male and female result from the intrinsic limits of our approach in terms of sensitivity and precision. In any case, there are no striking sex differences in the 3’UTR isoforms of Olfr mRNAs, for any of the parameters we examined.

## Discussion

### A new landscape of 3’UTRs for Olfr mRNAs

#### Extensive alternative polyadenylation of Olfr mRNAs

The spectacular development of high-throughput sequencing during the past decade uncovered the production of alternative mRNA isoforms from a growing number of genes. It turned out that alternative polyadenylation is widely used in biological systems, and even more particularly in the nervous system [61]. Whereas previous RNA-Seq analyses already identified multiple isoforms through Cufflinks annotation for a large number of Olfr mRNAs, these multiple isoforms were mostly the result of alternative transcription start sites and alternative splicing in the 5’UTR [26, 27]. Using a dedicated pipeline, we uncovered extensive APA for Olfr mRNAs in the adult mouse olfactory mucosa (76.6–78.4% of the annotated Olfr genes), allowing us to document a novel level of diversity of Olfr gene products. Importantly, we confirmed experimentally for the first time, for two representative Olfr genes, the repertoire of 3’UTR isoforms predicted by our computational pipeline. We further showed that 3’UTRs of Olfr mRNAs are rarely subject to alternative or optional splicing, an observation in line with the very low number of exons detected in the STAR output in the 3’UTRs of Olfr mRNAs (Additional file 6: Table S3). Thus, we demonstrate that the diversity of the Olfr mRNAs in terms of 3’UTRs is essentially the result of APA events.

#### Longer Olfr 3’UTRs than previously proposed

In a recent study based on the use of Cufflinks, Shum and colleagues reported that the Olfr mRNAs have exceptionally short 3’UTRs, with a median length of 773 nt [27]. In the present study, we clearly disprove this view, thanks to the use of a more appropriate method for 3’UTR analyses. As compared to Cufflinks, IsoSCM not only allows much better detection of APA, but also improves the detection of full-length UTRs (e.g. by merging transcript fragments separated by small gaps) [41]. We have established that the median length of single 3’UTRs is 1.0–1.2 kb (depending on the dataset considered). While the median length of proximal 3’UTRs ranges from 0.6 to 0.8 kb, the median length of the distal 3’UTRs reaches 1.9 to 2.2 kb in the adult OM. Thus, the 3’UTR length of the Olfr genes falls in the range of the 3’UTR length reported for other mouse genes in various studies [32, 62, 63]. Among the Olfr genes with multiple 3’UTR isoforms (in the male_IS2014 dataset), we noticed that the shortest isoform (pUTR) is generally the most highly expressed (true for 95% of Olfr genes with 2 or more 3’UTR isoforms; Additional file 3: Table S2). Thirty-eight % of the Olfr genes with multiple 3’isoforms have distal isoforms of quantitative importance (i.e. belongs to the P3-P4 quantitative profiles vs. P2). The fact that pUTRs are significantly shorter than sUTRs argues for a specific proximal UTR population, distinct from single UTRs.

### Validation of our pipeline

#### Comprehensiveness of our 3’UTR annotations

In their original RNA-Seq analysis, Ibarra-Soria and colleagues [26] reported the annotation of 913 Olfr full-length gene models. Focusing on the identification of 3′ ends, our pipeline allowed the accurate annotation of only 600–800 of Olfr genes, depending on the dataset (corresponding to 53.8–71% of the expressed Olfr genes). Consequently, our description of the 3’UTR landscape for Olfr mRNAs is not comprehensive. The analyses of the 4 datasets presented here show many substantial variations in the number, length and/or relative abundance of 3’UTR isoforms for individual Olfr genes (Additional file 3: Table S2; Additional file 9: Figure S6 and Additional file 10: Table S4 for the pilot genes), most probably for the following reasons. Even with the same genetic background (C57Bl6) and similar age of the 2 groups of adult males or females, unavoidable biological differences may arise in independent same-sex datasets. In addition, some technical differences such as sample preparation (stranded vs. non-stranded) and Illumina sequencing system (2000 vs. 2500, with respective read length of 75 vs. 100 nt) are likely producing differences in the 3′ end annotations between the 2 experimental series. Overall, some intrinsic features of the Olfr gene family and their regulated expression limit the depth of our analysis, as well as caveats of our pipeline: 1) low coverage in 3′ regions as compared to the requisite of the IsoSCM method; 2) heterogeneity in coverage in 3′ regions; 3) biases due to the 100-nt precision (such as those discussed below for very short 3’UTRs); and 4) interference between contiguous genes (such as improper fusions between 2 Olfr gene products).

##### Coverage in 3′ regions

One major limitation of our study is the relatively low abundance of Olfr mRNAs within the entire olfactory mucosa, which may affect both the number of annotated genes and the precision of the annotation generated by IsoSCM. This is illustrated by the fact that the Olfr relative expression level is higher in the Ibarra-Soria 2017 experiment than in the 2014 one, and the resulting numbers of annotated Olfr genes are higher in the 2017 Ibarra-Soria et al. analysis (Additional file 8: Figure S5). Moreover, the RNA-Seq read coverage is not uniform due to experimental biases [41]. In particular, a positional bias has been observed, generating a lower coverage in 3′ regions of the mRNAs than in upstream regions [64]. Consequently, most of the distal UTRs are under the minimal expression threshold of 200 reads/kb required for a 100-nt precision of the IsoSCM annotation tool (with a 10% false positive rate) [41]. Accordingly, we observed that the dUTRs are less conserved at a fixed rank between the 2 male datasets than sUTRs or pUTRs, and that the conservation decreases with the rank of the dUTRs (dUTR1 > dUTR2 > dUTR3 > dUTR4). Moreover, non-coding sequences show an increased frequency of repeat sequences as compared to CDSs, producing gaps in the UTR coverage [65]. Thus, a large part of the variability observed between the datasets may be attributed to this heterogeneous and low coverage in 3′ regions, even lower in distal isoforms.

##### Very short 3’UTRs

In our pipeline, we excluded all 3′ ends occurring within the CDS, but we retained 3′ ends located downstream and very close to the stop codon of Olfr genes (3’UTR length < 100 nt). Some of these very short 3′ ends could actually occur within the CDS given the 100-nt precision window, or could be artifacts due to a dissimilar coverage between the CDS and the downstream sequence. It should be further noted that the relative abundance of these very short 3’UTRs may be inaccurate, due to the calculation method used. Therefore, the reliability of these very short 3’UTRs (representing 11.9% of the Olfr genes annotated in both male datasets and 12.5% for both female datasets) is probably not as strong as that of longer ones. Accordingly, only half of these very short 3’UTRs are conserved in inter-experiment comparisons (51.3% for males, 49.3% for females), whereas 75% of the proximal or unique 3’UTRs (without length threshold) are conserved in these comparisons.

##### Fusion transcripts between adjacent Olfr genes

Along the development of our pipeline dedicated to Olfr 3’UTRs’ characterization, we obtained transcripts corresponding to the fusion of 2 Olfr genes (18–61 Olfr genes concerned depending on the dataset considered; Additional file 2: Figure S1E-H,). Chimeric mRNAs have been widely studied in pathological contexts, such as cancer [66], but their occurrence in physiological situations is a matter of debate, and detection of these fusions is one of the challenges of RNA-Seq analyses. They are either technical artifacts (e.g. template switching of reverse transcriptase), or trans-splicing products and read-through events, especially in regions with higher gene density [67, 68]. Olfr genes present many distinctive features including an expression pattern essentially restricted to OSNs, the rare possibility of 2 or more Olfr co-expression in one developing OSN [69–71] and high gene density as compared to the mean gene density in the mouse genome. Thus, the fusions observed in Olfr transcript annotation could result from: 1) technical artifacts, 2) continuity in the coverage between independent transcripts from two adjacent Olfr genes, or 3) genuine chimeric RNAs. Evidence for such chimeric Olfr1030 and Olfr1031 RNA was recently obtained by single-cell RNA-Seq analysis [69]. However, in the case of the continuous coverage in the 3′-3′ inter-CDS region of Olfr1508 and Olfr1509 genes, we favor the hypothesis of the coverage superposition of 2 independent transcripts (Fig. 5a). Since these genes are on opposite strands, we can exclude a chimeric RNA leading to co-expression of both proteins. In our pipeline, we restricted trans-splicing possibilities (setting a maximal size for introns) and we limited the merge_radius parameter to limit the creation of presumably artifactual fusions. Then, we decided to discard all fusion events involving Olfr 3’UTRs from our analysis. Consequently, we decreased the comprehensiveness of our study, and over-estimated the relative abundance of the remaining isoforms. For example, the 2 more distal 3’UTRs of Olfr1509 were experimentally validated by RL-PAT but were not annotated through our pipeline (Fig. 5b-d). To go further, the discrimination between false positive and true positive fusion events would require a targeted and extensive experimental validation, or a dedicated study of single-cell RNA-Seq datasets.

#### Canonical polyA signals at 3′ ends

From our study, it emerged that only 66.8 to 69.7% of 3’UTRs (without merging the neighboring 3′ ends) can be generated by the use of canonical PASs (AAUAAA and AUUAAA) placed in the [− 100;+ 100] precision window. This raised the question of the validity of those other 3’UTRs generated by our pipeline, having no correlation with well placed canonical PASs. In fact, these percentages perfectly fit with those previously characterized in other tissues. In mouse cDNA/EST, about 75% of polyA sites are associated with AAUAAA or AUUAAA hexamers [60]. In the brain, 40 to 70% of the 3′ ends are associated with these hexamers, depending on the subsets of 3′ ends analyzed [62]. Additionally, up to 16 variant hexamers have been characterized in mouse [72]. In the mouse retina analyzed at different developmental stages, 85% of the 3′ ends are associated with the AAUAAA hexamer or the most common 12 variants in human [33]. A residual part of the 3’UTRs is associated with neither canonical PASs nor variants, and there is growing evidence for functional non-canonical PASs [73]. Some Olfr 3′ ends may result from the use of either a variant canonical signal or a non canonical signal, as this is most probably the case respectively for Olfr16 3’M and Olfr1507 XS 3’UTRs (Additional file 10: Table S4). Thus, we can conclude that Olfr genes, like other genes and with roughly the same proportions, probably use canonical, variant and non-canonical PASs for generating diverse 3’UTRs.

### Functional significance of Olfr APA

#### Diverse APA profiles of Olfr mRNAs

Using our computational pipeline, we showed that only 21–23% of the Olfr genes unambiguously identified in the present study produce a single detectable 3’UTR. Of the remaining Olfr genes with multiple 3’UTR isoforms, 2/3 show a main isoform (relative abundance > 80%), whereas 1/3 show 2 or more main isoforms. Thus, APA seems to be widely used to generate alternative 3’UTR isoforms within the large Olfr gene family, and the extent to which APA is used is apparently differently regulated depending on the Olfr gene considered. We were surprised to notice that 3 Olfr genes belonging to the same OR sub-family, Olfr1507, Olfr1508 and Olfr1509 [14], show diverse APA profiles (Figs. 3 and 5). The Olfr1507 and Olfr1508 genes were recently duplicated from an ancestral gene, with 92% identity in the coding regions [74]; however, their 3’UTR isoforms differ in number, length and relative abundance profiles. Since Class I and Class II Olfr genes show no difference in APA, the determinants for this diversification of APA profiles between subsets of Olfr genes remain to be identified.

We cannot exclude that the Olfr genes for which we detected a single 3’UTR isoform in the present study may be subject to APA at other developmental stages, or under different physiological circumstances (i.e. depending on olfactory stimuli). Likewise, it remains to be determined if an Olfr characterized by one of the 4 profiles (P1, P2, P3 or P4), defined here depending on the relative expression levels of the multiple isoforms, may switch to another profile under different circumstances. Our study focused on adult whole olfactory mucosa and a choice of different physiological situations should now be investigated. Lengthening of 3’UTR by APA during mouse embryonic development has been reported [32]. Extension of 3’UTR occurs in a tissue-specific manner in mouse neural tissue and along the differentiation of neurons from pluripotent ES cells [62, 75]. In Drosophila, lengthening of 3’UTR was observed during the development of the central nervous system [31]. More recently, extension of 3’UTR by APA was demonstrated during the mouse retinal development [33]. The neuronal activity is also critical for the 3’UTR length regulation by APA [39, 76], as well as the pathological or lesion/regeneration contexts [77]. Even the relatively rare alternative isoforms may indeed be of physiological importance in the olfactory sensory neurons, if they have a specific role or subcellular compartmentalization (see below) as compared to the main isoform(s).

#### No clear evidence for sexual dimorphism in Olfr 3’UTRs

We took advantage of the availability of similar male and female datasets to investigate possible sex-specific difference in the repertoires of 3’UTRs of Olfr mRNAs expressed in the adult olfactory mucosa. We observed no sex-specific introns and no global differences in APA, in terms of APA distribution, length of single proximal and distal 3’UTRs, or on the quantitative 3’UTR isoform profiles of Olfr mRNAs between male and female. Moreover, 3 out of the 4 sex-specific pUTRs identified belong to the very short 3’UTR category (< 100 nt for at least one of the datasets), raising the question of their reliability. Therefore, while we cannot rule out the possibility of sex-specific 3’UTR isoforms for some individual Olfr genes, if these isoforms exist, they would involve a small number of these genes. It has already been shown that the Olfr expression level was highly similar between males and females [26], except for the Olfr1347 receptor. This particular Olfr is not annotated in 3 out of the 4 datasets, and the analysis of the coverage argues in favor of a conserved sUTR for Olfr1347 between males and females. Thus, it seems very unlikely that sex-specific 3’UTR isoforms of Olfr mRNAs play a role in sexually dimorphic behaviors linked to odor detection; these behaviors were rather proposed to be due to sex-specific handling of olfactory information by central circuits [78].

#### Functional role for Olfr 3’UTR isoforms

The fact that the distal 3’UTRs of Olfr mRNAs are much longer than the proximal 3’UTRs opens new avenues for the discovery of regulating sequences or structures specific to the alternative 3′ regions, such as sequences dedicated to the regulation of the mRNA metabolism, translation or subcellular localization. Interestingly, several mRNAs transported in axons are specific alternative isoforms with a long 3’UTR, which undergo regulated local translation [34–38, 79]. There is compelling evidence for the localization of Olfr mRNAs in OSN axons, and for their local translation [6, 7, 80], and our present work demonstrates that they exhibit alternative polyadenylation. Thus, the identification of any axon specific 3’UTR isoforms of the Olfr mRNAs should be investigated. Additionally, it will be of critical interest in future studies to determine if all multiple 3’UTR isoforms generated by a given Olfr gene are co-expressed by the same OSNs, or if the different isoforms are differentially expressed by different neurons. This might well be the case, given the fact that, because of the continuous neurogenesis occurring in the OM, the adult OM contains both immature and mature OSNs. Because of the known regulation of APA occurring as a system develops, it is possible that immature and mature OSN expressing the same Olfr may express different 3’UTR isoforms of the corresponding Olfr mRNA.

## Conclusions

The family of odorant receptor genes is the largest gene family in mammals, which provides a unique opportunity to study the diversity and variations of 3’UTRs for a large number of phylogenetically closely related genes within a given species. To study the 3’UTR isoforms of the Olfr genes, we developed a new dedicated pipeline and applied it to RNA-Seq datasets obtained from adult mouse olfactory mucosa of both sexes. The annotations obtained were further validated by multiple experimental approaches on pilot genes, and canonical or variant polyA signals were found associated with these 3′ ends identified in silico. Whereas most of the Olfr genes show variations in 3’UTR length, we detected only rare splicing events downstream stop codons. Thus, we demonstrated that a large number of Olfr genes produce multiple 3’UTR isoforms, mostly generated through alternative polyadenylation. Strikingly, the diversity in 3’UTRs for the Olfr gene family seems similar to the diversity observed for the whole genome. This alternative polyadenylation is dependent neither on the sex of the animals, nor on the Class I vs. Class II Olfr gene subfamilies. Thus, the determinants for the APA regulation of Olfr genes remain to be investigated.

In this study, we chose to explore publicly available RNA-Seq datasets from adult mouse whole olfactory mucosa. While RNA-Seq became the reference method for high-throughput characterization of the transcriptome, alternative dedicated methods now exist to characterized more specifically the 3′ ends of mRNAs, such as 3′-seq [81] or PAPERCLIP, even at a cell-type specific level [82, 83]. However, the pipeline used in the present study could be extended to different situations for which RNA-Seq data is either available or easy to obtain, such as: 1) subpopulation of cells in the olfactory mucosa (e.g. FACS-sorted OSNs from OMP-GFP mice); 2) whole olfactory mucosa from animals of different ages or differently exposed to olfactory stimuli; 3) mouse tissues where Olfr genes are ectopically expressed; 4) olfactory mucosa from other species (e.g. humans). These analyses are clearly beyond the scope of this paper, but they will provide valuable information regarding the regulation of Olfr APA in various circumstances, hence providing hypotheses regarding the functions of Olfr alternative 3’UTR isoforms. Interestingly, the 3’UTR length of odorant receptor mRNAs was shown to be highly variable in human (2.8 ± 2.0 kb for a subset of genes annotated with Cufflinks) [84], and we anticipate that the alternative polyadenylation of odorant receptor mRNAs could be a common feature in Mammals. Our prediction is that alternative 3′ regions may include specific sequences or structures important for the different roles played by the odorant receptors in the development and function of the olfactory system.

## Methods

### RNA-Seq analysis

#### Hardware and software

This pipeline was constructed on a GNU/Linux operating system (Ubuntu 14) eight cores and 96 GB of RAM, as well as 2 TB of hard drive space.

The computational pipeline is a complete bash shell and perl scripts using internal implementation or external software for analyzing RNA-Sequencing, available at https://github.com/doulazmi/Olfr_RNAseq. The analysis steps are expressed in terms of parsing input files to output files as part of the overall workflow (Fig. 2).

#### Data sets analyzed

Sequencing fastq raw files with RNA-Seq PRJEB1365 [26] and PRJEB5984 [40] were downloaded from the European Nucleotide Archive (ENA).

#### Preparation of reference genome

The reference genome for mouse (mm10) was downloaded in compressed FASTA format from UCSC’s genomes (University of California, Santa Cruz). Furthermore, the comprehensive gene annotation for mm10 was downloaded in the GTF format from GENCODE (Release M14; GRCm38.p5).

#### RNA-Seq data processing and alignment

Paired-end read files were aligned to version mm10 of mouse reference genome and transcriptome using STAR 2.5.1b [85] with the following flags -runThreadN 7 --outSAMtype BAM SortedByCoordinate --outSAMstrandField intronMotif --outFilterIntronMotifs RemoveNoncanonical --alignIntronMax 23,000.

Threshold for maximal intron size was set to 23 kb, since the largest coherent 5′ intron described for an Olfr in GRCm38.p5 reaches 22.628 pb (Olfr872).

STAR output files in BAM format were sorted and indexed with SAMtools 1.5 [86].

#### Preparing a mask file in bed format restricted to the Olfr loci in mouse

In order to reduce computational time, and to limit the impact of non-Olfr genes on Olfr transcript assembly, we applied a mask restricted to Olfr genomic regions before IsoSCM and Cufflinks assemblies.

Starting with the GTF file downloaded from GENCODE (GRCm38.p5), and using an in-house perl script, we extracted Olfr CDS positions based on Olfr name. Consequently, most of Olfr pseudogenes (without known CDS) were discarded. To take into account the possibility of alternative CDSs, we defined CDS position for each gene as the maximal distance from start to stop codons of all isoforms. We then calculated the distance to upstream and downstream CDS for each Olfr. For Olfr190 and Olfr912, we retained the 2 distinct loci annotated for each of these genes. In databases, Olfr643 presents an alternative transcript overlapping with multiple Olfr genes (ENSMUST00000138055.1); since it was undergoing non-sense mediated mRNA decay, this particular transcript was discarded from our list. As Olfr56 shows multiple transcripts in databases (some overlapping with multiple genes due to very large introns), we kept the whole region between Olfr1396 et Olfr1394, including the most probable Olfr56 CDS (CCDS24597.1 Chr11:49134290–49,135,141) and eliminating the longer Olfr56 transcripts.

We defined Olfr genomic regions as the regions including a single or consecutive Olfr CDSs from the proximal end of the closer upstream non-Olfr gene to the proximal end of the closer downstream non-Olfr gene. As Olfr161 has no upstream non-Olfr gene on chromosome 16, the upstream inter-CDS distance was arbitrarily set to 25 kb. Gene density in Olfr genomic regions was calculated as the distance from the downstream end of the non-Olfr CDS upstream of the first Olfr CDS in the Olfr genomic region to the upstream end of the non-Olfr CDS downstream of the last Olfr CDS in the Olfr genomic region divided by the number of Olfr CDSs in this Olfr genomic region.

Additionally, we included in the dataset genes that were either overlapping with Olfr genes (and accepted to be isoforms of the corresponding Olfr in Ensembl) or defined as Odorant Receptor genes. The predicted gene Gm20715 (undergoing non-sense mediated mRNA decay) was removed and upstream inter-CDS distance for Olfr1344 was calculated using the Smim17 gene [70]. Moreover, given the proximity of Olfr94 with the Ubd gene, its 3’UTR annotation was discarded. Finally, the Olfr mask included 103 Olfr genomic regions gathering 1181 Odorant Receptor genes (including 1159 Olfr genes), and covered more than 35 Mb (Additional file 1: Table S1).

#### Masking the alignments files

The alignments BAM files (sorted and indexed output of STAR) were masked with SAMtools to keep only the reads inside the mask Olfr regions.

#### Transcript assembly with cufflinks

Transcripts were assembled from the mapped and masked fragments sorted by reference position. Fragments were first divided into non-overlapping loci, and each locus was assembled independently of the others using the Cufflinks (2.2.0) assembler with the default parameters [87].

#### Transcript assembly with IsoSCM (isoform structural change model)

IsoSCM assembled aligned and masked reads into a splice graph, identified nested terminal exons boundaries using the constrained segmentation procedure, and reported the resulting models in GTF (Additional files 11, 12, 13 and 14) [41]. We considered 2 parameters of IsoSCM in order to maximize the number of 3’UTR isoforms detected and find the optimal balance between fragmentation of long 3’UTRs and improper fusion of consecutive Olfr genes.

First, we tested a large range of values for the segmentation parameter min_fold, which represents the minimum fold change between neighboring segments: 0.1, 0.2, 0.3, 0.4, 0.5 (default setting), 0.6, 0.7, 0.8 or 0.9. We anticipated that a higher min_fold might allow the detection of even rare longer 3’UTRs. Indeed, increasing min_fold up to 0.8 systematically increased the total number of 3’UTR isoforms identified as compared to the 0.5 default value, thus increasing the comprehensiveness of the annotation (Additional file 2: Figure S1A-D for clarity, results are indicated for min_fold 0.5 and 0.8 only).

Second, the assembly parameter merge_radius, which represents the maximum gap length for merging fragments, was set at different values: 50, 100 (default value), 200, 300, 400 or 500 nt. Since merge_radius correlates with improper fusion of consecutive Olfr genes (Additional file 2: Figure S1E-H), increasing merge_radius over 300 nt appeared unfavorable. Increasing merge_radius to 200 nt increased the total number of 3’UTR isoforms identified as compared to default or lower values, due to fragmentation prevention (Additional file 2: Figure S1A-D, compare 200 nt and 100 nt). We therefore set the merge_radius at 200 nt and we paid attention to discard the 18 to 61 fused transcripts (i.e. including at least part of the 5’UTR and/or CDS of a neighboring Olfr, Additional file 2: Figure S1E-H) that remained, simply because of the very high gene density in Olfr genomic regions (up to 67.1 CDSs/Mb, Additional file 1: Table S1). These 3’UTR isoforms due to fused transcripts, for which attribution was ambiguous between 2 adjacent Olfr genes, were discarded (Fig. 2, step 5).

Interestingly, the optimized parameters defined above were confirmed on the subset of 3′ ends obtained after merging the 3′ ends distant of less than 100 nt (Additional file 2: Figure S1A-D, 100-nt precision subset, grey labels) and the subset of PAS-associated 3′ ends (Additional file 2: Figure S1A-D, PAS-associated subset, orange labels). Thus, we definitely set min_fold and merge_radius parameters to 0.8 and 200 respectively.

Last, the visualization parameter max_isoform, which represents the maximal number of isoforms at a locus, was set to 20 (default value = 5), to avoid skipping of gene isoforms due to multiple variants already known in 5′.

#### Transcript annotations

Models GTF files generated in the previous step were converted to bed files with gtf2bed tool then scanned with Bedtools 2.23 intersect function [88] to detect presumably artifactual fused transcript overlapping one Olfr and adjacent non-Olfr genes.

We matched curated transcripts with the position in BED format of all the Olfr genes to retrieve the list of transcripts that are uniquely overlapping to each Olfr gene.

#### Annotation of 3’UTR isoforms

From the GTF file GRCm38.p5, we extracted Olfr stop codon positions based on Olfr name using in-house perl script**.** The list of stop positions was manually curated for ambiguous multiple stops per Olfr for Olfr93 and Olfr643: stop codons of transcripts thought to undergo nonsense mediated decay were discarded.

In-house perl scripts matching Olfr stop codon coordinates against 3p_exon transcripts from assembled BED and GTF files were used to identify and characterize the Olfr 3’UTR isoforms. 3’UTR isoforms of the same gene were merged when 3′ ends were distant of less than 100 nt (precision window).

#### Detection of polyadenylation signals

At each position in a 100-nt window upstream of and downstream from annotation of 3′ UTR ends, an in-house perl script with internal implementation or the polyADQ external program [89] was used to detect the AAUAAA/AUUAAA canonical PASs. Variant PASs among the 16 most common non canonical hexamers [72] were manually detected in a 100-nt window upstream cleavage site for individual cases of 3′ end lacking a canonical PAS.

#### 3′ intron detection from RNA-Seq

To better characterize introns occurring in 3’UTRs, we used the regtools junctions extract function to get splice junctions from alignments BAM files. Then, we classified reported introns on the basis of the best score and the overlap with modeled 3’UTR isoforms.

#### Quantification of the 3’UTR isoforms

We considered that any Olfr gene with at least one fragment mapping in the CDS is expressed. Ibarra-Soria and colleagues previously demonstrated that expression estimations by RNA-Seq as Fragments Per Kilobase of exon per million fragments mapped (FPKM) are confident in the OM [26].

Bedtools coverageBed was then used to compute the genome coverage from the alignment based on the read depths at each base position followed by the output processing using an in-house perl script. The number of reads per kb in each 3’UTR isoform was counted.

For all alternative isoforms of the same Olfr gene, we estimated the relative abundance of each isoform. First, segments were defined as the regions between stop codon and the most proximal 3′ end or between 2 adjacent 3′ ends. The abundance of each segment was calculated as the difference between the total counts (bedtools output) upstream its end and the total counts upstream its start, divided by the length of the segment. Then, relative abundance of an isoform was calculated as the ratio of the difference of abundance of 2 adjacent segments (abundance of the most distal segment of the quantified isoform minus abundance of the downstream segment) and the total coverage of the most proximal segment for this Olfr gene.

#### Statistical analysis

All manipulations and statistical analyses were implemented with R (3.3.0). The statistical analysis focused on annotated Olfr genes with at most 5 isoforms. Normality in the variable distributions was assessed by the Shapiro-Wilk test. Furthermore, the Levene test was performed to probe homogeneity of variances across groups. Variables that failed the Shapiro-Wilk or the Levene test were analyzed with nonparametric statistics using the one-way Kruskal–Wallis analysis of variance on ranks followed by Nemenyi test post hoc and Mann–Whitney rank sum tests for pair-wise multiple comparisons. Variables that passed the normality test were analyzed by means of one or two-way ANOVA followed by Tukey post hoc test for multiple comparisons or by Student’s t test for comparing two groups. Categorical variables were compared using Pearson’s Chi-squared test or Fisher’s exact test.

The linear regression analysis was used to estimate the fitting expression level between the different datasets. To define different relative-abundance profiles for Olfr 3’UTR isoforms, an unsupervised approach (hierarchical cluster analysis) based on principal component analysis (PCA) was used. The PCA was performed based on the relative abundance and length of alternative isoforms. Based on the PCA results, we applied hierarchical clustering to determine automatically the different relative-abundance profiles described in the results section. All PCA was performed using FactoMineR package.

A *p* value of < 0.05 was used as a cutoff for statistical significance. Results are presented as the mean ± SD or medians, unless otherwise stated. The statistical tests are described in each figure legend.

#### Visualization of data

All the figures were automatically generated with *ggplot2* package using R scripts written for this purpose. Furthermore, Venn diagrams were generated using the *VennDiagram* R package to visualize the Olfr genes that were annotated in multiple datasets.

### RNA extraction

Six adult (8–12 weeks) male C57Bl6 mice were obtained from a commercial source (Janvier labs, Saint Berthevin, France). They were sacrificed by cervical dislocation. The whole olfactory mucosae were then immediately dissected (3 samples for northern blot applications; 3 samples for RL-PAT or 3’RACE analyses). Tissues were either frozen at − 80 °C before homogenization, or directly homogenized in 1 mL QIAzol lysis reagent (QIAGEN) with a rotor-stator homogenizer and incubated 5 min at room temperature. After 200 μL chloroform addition, samples were incubated 2 min at room temperature and centrifuge for 15 min 12,000 rcf at 4 °C. Total RNAs were isolated from aqueous phase using either the RNeasy Mini kit (QIAGEN) or the Nucleospin RNA kit (Macherey-Nagel) following manufacturer instructions, with (for northern blot applications) or without (for RL-PAT or 3’RACE analyses, see below) on-column DNase treatment. Concentrations were measured with the Nanovue spectrophotometer (GE Healthcare).

### RL-PAT (RNA ligation mediated PolyAdenylation test)

RNA were added with 7–8 pmol of P1 oligonucleotide (Table 2) per μg of RNA and denatured for 5 min at 75 °C prior to ligation 30 min at 37 °C with T4 RNA ligase in appropriate buffer added with 1 mM ATP and 0.1 μg/μL BSA (Fermentas, Thermo Fisher Scientific). Ligation was stopped by thermal denaturation 10 min at 70 °C. 10 μL of ligation products were mixed with 2.3 pmol of P’1 oligonucleotide (Table 2) and 8 mmol desoxyribunucleotides triphosphate (dNTPs) and denatured for 5 min at 65 °C prior to reverse transcription 30 min at 55 °C with Maxima reverse transcriptase in appropriate buffer added with 1 u/μL Ribolock RNase inhibitor (Fermentas, Thermo Fisher Scientific). For negative controls without reverse transcription, the enzyme was omitted. Reverse transcription was stopped by thermal denaturation 5 min at 85 °C. Usage of identified polyadenylation sites was tested by PCR amplification using P’1 as a reverse primer and site-specific P2 forward primers (Table 2) with HotStarTaq DNA polymerase (QIAGEN). PCR products were visualized after electrophoresis on agarose gels, TA cloned in pCR4-TOPO (TOPO TA cloning kit for sequencing, Invitrogen, Thermo Fisher Scientific) following manufacturer instructions and Sanger sequenced (Genewiz). Reference repeat masked sequences of the Olfr genes were downloaded from Ensembl [45].

**Table 2:** Oligonucleotides used in RL-PAT experiments

General primers	Primer sequence		
P1	5′-GGTCACCTTGATCTGAAGC-3’		
P1’	5′-GCTTCAGATCAAGGTGACCTTTTT-3’		
Gene specific primers	Forward primer sequence (P2)	PolyA site tested	Position (nt after stop)
Olfr1507	5′-TGACATGACAACATTTCATTCTGA-3’	XS	69
	5′-ATCTGTATTCACTCAAAGAGTTTAGAGTTT-3’	S	1224
	5′-GATCTCCTGAGCCATCAACTATCA-3’	M	1986
	5′-TGGGCCTTCCTAAATTCTTAAATA-3’	L	3993
Olfr15	5′-ATCCAGCAACTGGTTTAGAACAAA-3’	S	788
	5′-AAGCTGGGGTTACAACTCAGTGAT-3’	M2	2698
Olfr1508	5′-TACTGTTTGCAATTGTGTGAATTT-3’	XS	2586
	5′-TGTAACACCTTGAATAGCTCAAAA-3’	S	2915
	5′-TCAAAGGGATTCAATAACTTCTTC-3’	M	4133
Olfr1509	5′-GTGCTTGTGTGATGTTGTGATAA-3’	XS	15
	5′-CTAGGTGTATAATGTTTGGGGTTT-3’	S	289
	5′-TGTTTTACTATCCATTGGCTTGAT-3’		348
	5′-CGATAGACAACTTGGAAGAGAACT-3’	M	1086
	5′-CTATCGTTCCAGTTAGGACTTCAC-3’	L	4347
Olfr16	5′-ACATGCTCATCAAATACGGTGTCT-3’	S	69
	5′-AAAGGCCCCACATTTCATTATTTA-3’	M	498

The general primers consist in a synthetic sequence P1 and its reverse complement P’1 with a (T)_5_ anchor [90]

### Northern blot

To generate probe templates, targeted regions of Olfr mRNAs were amplified by PCR from genomic DNA or olfactory mucosa complementary DNAs (C57Bl6 mouse) and cloned in pBlueScript (Table 3). Plasmids were linearized using Fast Digest restriction enzymes (Fermentas, Thermo Fisher Scientific) and purified with the Nucleospin gel and PCR cleanup kit (Macherey-Nagel). Probes were synthesized by in vitro translation using DIG (digoxygenin) RNA labeling mix and T3 or T7 RNA polymerases (Roche), and purified on Illustra ProbeQuant G-50 Micro Columns (GE Healthcare). Concentrations were estimated following electrophoresis on agarose gels. Probes were denatured 5 min at 95 °C prior to hybridization.

**Table 3:** Description of the probes used in northern blot experiments

Gene name	Probe name	Target region description	Size of target region (nt)
Ensembl transcript			
Olfr1507	5’e	from 46 to 619 of the 5′ non coding exon 1	574
ENSMUST00000206062.3	5’i	from 1949 to 2527 of the 5′ intron 1	579
	CDS	from 555 of CDS to 130 after stop	518
	3′S	from 872 to 1282 after stop	411
	3’M	from 1761 to 2384 after stop	624
Olfr15	CDS	CDS w/o stop	936
ENSMUST00000080917.1	3’M	from 1238 to 2150 after stop	913

Total RNA (600 ng/lane in 2:1 RNA sample loading buffer w/o ethidium bromide (SIGMA)) was separated on an agarose 1% formaldehyde 2% gel in MOPS buffer (SIGMA). Molecular weight markers used were RNA MW marker I, DIG-labeled 0.3–6.9 kb (Roche) and RiboRuler High Range RNA ladder (Thermo Fisher Scientific). The gel was soaked 2 × 15 min in 20X SSC before overnight capillary transfer on a nylon membrane, positively charged (Roche) using 20X SSC as transfer buffer. RNA was fixed on the membrane by Ultra-Violet crosslinking (UV Stratalinker 120 mJ). The membrane was washed with water, air dried and cut for separated hybridization of lanes with different probes. The membranes were then prehybridized in prewarmed hybridization buffer (DIG Easy Hyb Granules Roche, 0.2 mL/cm^2^) 30 min at 68 °C in sealed bags placed in rotating tubes, and further hybridized over night at 68 °C with DIG labeled RNA probes (100 ng/mL, 0.07 mL/cm^2^). Hybridized membranes were washed under agitation successively 2 × 5 min in 2X SSC 0.1% SDS at room temperature; 2 × 15 min in 0.1X SSC 0.1% SDS at 70 °C; 1 × 2 min in Maleic Acid Buffer (0.1 M Maleic acid (SIGMA), 0.15 NaCl pH 7.5) with 0.3% Tween-20. The membranes were blocked for 30 min in Blocking Solution (Blocking reagent, Roche, diluted in Maleic Acid Buffer) and incubated for 30 min in Anti-DIG-AP Fab fragments 1/10,000 (Roche) in Blocking Solution at room temperature under agitation. The membranes were washed under agitation successively 2 × 15 min in Maleic Acid Buffer with 0.3% Tween-20; 1 × 3 min in 0.1 M Tris-HCl 0.1 M NaCl pH 9.5 prior to revelation with CDP-Star reagent (Roche). Signal was acquired by exposition to Amersham Hyperfilm ECL films (GE Healthcare). Whole blots are shown in Additional file 15: Figure S7A-C, Additional file 15. Signal specificity was tested by parallel hybridization with antisense and sense probes (Additional file 15: Figure S7B-C, Additional file 15: Figure S7).

### 3’RACE (3′ rapid amplification of cDNA ends)

10 μg of total RNA were incubated with Turbo DNase (Invitrogen, Thermo Fisher Scientific) following manufacturer instructions. 2 μg of DNA-free RNA were denatured with 200 pmol of GR3dT oligonucleotide (Table 4) and 0.33 mmol dNTPs for 5 min at 65 °C prior to reverse transcription 30 min at 50 °C with Maxima reverse transcriptase in appropriate buffer (Fermentas, Thermo Fisher Scientific). Reverse transcription was stopped by thermal denaturation 5 min at 85 °C. 3’UTRs were then amplified by 2 successive PCR runs with Phusion DNA polymerase (Thermo Fisher Scientific): 1) using GR3-first as a reverse primer and Olfr1508-first as a gene-specific forward primer designed 1 kb downstream of the Olfr1508 stop codon, 2) using GR3-nested and Olfr1508-nested primers. PCR products were visualized after electrophoresis on agarose gels, TOPO cloned in pCR4-Blunt-TOPO (Zero Blunt TOPO PCR cloning kit for sequencing, Invitrogen, Thermo Fisher Scientific) following manufacturer instructions and Sanger sequenced (Genewiz).

**Table 4:** Oligonucleotides used in 3’RACE experiments

General primers	Primer sequence			Step
GR3-dT	5′-GCTGTCAACGATACGCTACGTAACGGCATGACAGTG(t)_18_(g,c,a)-3’			RT
GR3–1	5′-GCTGTCAACGATACGCTACGTAACG-3’			First round PCR
GR3–2	5′-CGCTACGTAACGGCATGACAGTG-3’			Second round PCR
Gene specific primers	Primer sequence	PolyA site tested	Position (nt after stop)	Step
Olfr1508–1	5′-AGCAGCAAACATTCCAAATTGAGGA-3’	M	1001	First round PCR
Olfr1508–2	5′-TGTGTTCACCTAAAGAGTTTAAGAC-3’		1145	Second round PCR

## Additional files

Additional file 1: **Table S1.** Olfr genomic regions included in the mask. An Olfr genomic region is defined by one Olfr CDS (isolated) or multiple consecutive Olfr CDSs bounded by an upstream and a downstream nonOlfr CDSs. Olfactory genomic region line (orange): # region, Number of Olfr genes (with a CDS), # chromosome, Upstream boundary (end of the upstream non-Olfr CDS), Downstream boundary (beginning of the downstream non-Olfr CDS), Region length (nt), Gene density (number of Olfr CDS divided by region length). Gene line: non-Olfr/Olfr flag, # chromosome, CDS upstream end, CDS downstream end, Gene name, Gene orientation, CDS length (nt). Comments: Isolated genes are indicated; As Olfr161 is the first gene on Chr16, the upstream boundary of the Olfr161 genomic region was arbitrarily established 25 kb upstream of the Olfr161 CDS. (XLSX 176 kb)

Additional file 2: **Figure S1.** Set up of IsoSCM min_fold and merge_radius parameters. A-D. Numbers of annotated 3’UTRs are higher when min_fold is set at 0.8 (solid triangles) as compared to default setting at 0.5 min_fold (empty triangles). Blue label = Total numbers of annotated 3’UTRs; grey label = numbers of 3’UTRs merging 3’ends under the 100-nt precision threshold; orange label = numbers of 3’UTRs matching at least one canonical AAUAAA or AUUAAA PAS in a [− 100;+ 100] window. E-F. Increasing merge_radius triggers higher numbers of Olfr genes showing annotation of chimeric exons between adjacent genes (discarded fusions). min_fold is set to 0.8 in E-F. (PDF 392 kb)

Additional file 3: **Table S2.** Alternative 3′ ends for Olfr genes annotated from the 4 datasets. The 3′ end positions and the relative abundances of the resulting 3’UTR isoforms were obtained using our IsoSCM workflow. For each individual dataset: Gene name, Length of the minimal CDS (covered by reads), Counts in the minimal CDS, Number of 3’UTR isoforms; for each utr (utr1-utr5): length and relative abundance (%RA); quantitative 3’UTR isoform profile (see Fig. 4 for the definition of the profiles). Additional column for the male_IS2014 dataset: pUTR vs. dUTR1 abundance flag (RA_utr1 > RA_utr2). Additional columns for comparisons: Very short flag (length < 100 in one of the dataset), ∆length (length difference between 2 datasets), conserved flag (∆length < 200), robust flag (ROBUST, conserved in all the comparisons of the 4 datasets), sex-specific flag (SEX-SPE, not conserved in same-sex comparisons but conserved between males and females in both experimental series), experience-specific flag (EXP-SPE, conserved in same-sex comparison but not conserved between males and females in both experimental series). (XLSX 1067 kb)

Additional file 4: **Figure S2.** Northern blot characterization of the intron-retaining transcript for Olfr1507. Total OM RNAs were separated on an agarose/formaldehyde gel and transferred onto a nitrocellulose membrane. The presence of Olfr1507 mRNAs was detected following hybridization with DIG-labeled antisense probes either in the 5′ exon (5’e), the 5′ intron (5’i), the CDS region (CDS), between the CDS and 3′S ends (3′S) or between the 3′S and 3’M ends (3’M) (see Table 3 for detailed probe description). The major isoform of the Olfr1507 mRNA is characterized by the absence of the 5′ intron that has been excised, and the presence of a short 3’UTR (≈3-kb dark band detected with 5’e, CDS and 3′S probes, not detected with 3’M or 5’i probes); the highest band (indicated with # in Fig. 3g) corresponds to an intron-retaining Olfr1507 mRNA bearing a short 3’UTR (> 7 kb light band detected with 5’e, 5’i, CDS and 3′S probes, not detected with 3’M probe). (PDF 1137 kb)

Additional file 5: **Figure S3.** Unbiased detection of APA for Olfr 3’UTRs in the 4 datasets. A, B, C, D. Expression levels of Olfr mRNAs (total counts in CDS) related to the number of 3’UTR isoforms per Olfr gene. One-way Kruskal-Wallis test (A: Chi square = 88.615, *p* < 0.0001, df = 4; B: Chi square = 144.89, df = 4, *p* < 0.0001; C: Chi square = 100.62, *p* < 0.0001, df = 4; D: Chi square = 119.06, df = 4, *p* < 0.0001), followed by Nemenyi test show no difference for expression levels between Olfr without APA (single 3’UTR) and Olfr with 2 or 3 3’UTR isoforms, demonstrating that, up to three 3’UTR isoforms, APA is detected independently of the expression level of the Olfr genes. However, annotation of more than 3 isoforms is biased by expression level, and we probably underestimate the number of 3’UTR isoforms (more than 3) for Olfr genes expressed at low level. (PDF 435 kb)

Additional file 6: **Table S3.** Optional introns detected in the Olfr 3’UTRs annotated with our IsoSCM workflow (male_IS2014 dataset). For each Olfr concerned: # chromosome, intron boundaries (Start/End), Gene name. (XLSX 14 kb)

Additional file 7: **Figure S4.** Unbiased detection of APA for Class I or Class II Olfr 3’UTRs in the male_IS2014 dataset A. Expression levels of Class I Olfr mRNAs (total counts in CDS) related to the number of 3’UTR isoforms per Olfr gene. One-way Kruskal-Wallis test (Chi-squared = 5.5307, df = 3, *p*-value = 0.136) shows no difference for expression levels between Olfr without APA (single 3’UTR) and others. B. Expression levels of Class II Olfr mRNAs (total counts in CDS) related to the number of 3’UTR isoforms per Olfr gene. One-way Kruskal-Wallis test (Chi-squared = 80.875, df = 4, *p* < 0.0001), followed by Nemenyi test show no difference for expression levels of Class II Olfr mRNAs with up to three 3’UTR isoforms, demonstrating that APA is detected independently of the expression level of the Class II Olfr genes. Annotation of more than 3 isoforms for Class II Olfr genes is biased by expression level, and we probably underestimate the number of 3’UTR isoforms (more than 3) for low expression Class II Olfr genes. (PDF 384 kb)

Additional file 8: **Figure S5.** Comparison of the subsets of annotated Olfr genes between the 4 datasets. A-D. Venn diagrams showing the numbers of Olfr genes with annotated 3’UTR(s) in 2 different datasets. The intersection of the 2 ellipses represent the common Olfr genes retrieved in the 2 datasets. E-H. Correlation of the expression levels between 2 datasets restricted to common Olfr with annotations in both datasets. The expression levels of Olfr genes are similar in the male and female datasets from each experiment (2014 or 2017) as previously shown in [26]. Pearson equation and coefficient were used to estimate the fitting level (*p* < 0.05) (PDF 570 kb)

Additional file 9: **Figure S6.** Alternative 3’UTR isoforms identified for 2 pilot genes in the 4 datasets. Graphical representation of the alternative 3’UTR isoforms annotated for Olfr1507 or Olfr15 in terms of 3’UTR length (left panels; blue bars) and relative abundance (right panels). (PDF 393 kb)

Additional file 10: **Table S4.** Alternative 3′ ends for pilot genes annotated from RNA-Seq data and RL-PAT experimental validation. The 3′ end positions and the relative abundances of the resulting 3’UTR isoforms were obtained using our IsoSCM workflow to process the 4 datasets from adult OM. The 3′ ends distant of less than 100 nt were merged as a single polyA site^a^. For each putative polyA site, predicted canonical PASs matching in a [− 100;+ 100] window from the RNA-Seq-deduced 3′ end are indicated^b^. Note that for Olfr1507 3’XL isoform, the closest canonical PAS is slightly outside the 100-nt precision window^c^. The cleavage site and polyA tail length were experimentally mapped using RL-PAT on adult male OM, further confirming the polyA site usage for most of the 3’UTR isoforms. XL site of Olfr1507, M1 and L sites of Olfr15 were not confirmed by RL-PAT since no specific forward primer could be designed due to repeated sequences upstream of these polyA sites^d^. For each confirmed polyA site, predicted canonical PASs, or variant hexamers when necessary, matching in a [− 100;0] window from the identified cleavage sites are indicated^e^. ND: not detected; NA: not available. (XLSX 39 kb)

Additional file 11: Annotations for Olfr genes using our IsoSCM pipeline on the male_IS2014 dataset. (GTF 1625 kb)

Additional file 12: Annotations for Olfr genes using our IsoSCM pipeline on the female_IS2014 dataset. (GTF 1294 kb)

Additional file 13: Annotations for Olfr genes using our IsoSCM pipeline on the male_IS2017 dataset. (GTF 2198 kb)

Additional file 14: Annotations for Olfr genes using our IsoSCM pipeline on the female_IS2017 dataset. (GTF 1757 kb)

Additional file 15: **Figure S7.** Whole northern blots and demonstration of the probe specificity for Olfr1507 and Olfr15 northern blot experiments. Whole northern blots correspond to Fig. 3g (A) and h (B) and Additional file 4: Figure S2 (C). The specificity of the antisense probes used in northern blots was confirmed by the absence of signals with the sense probes for Olfr1507 (B-C) or Olfr15 (A). See the corresponding figure legends and Table 3 for the detailed description of the probes. (PDF 363 kb)

## Data Availability

All data analyzed during this study are included in these published articles [26, 40] and are available in the European Nucleotide Archive (ENA) repository (https://www.ebi.ac.uk/ena/data/view/PRJEB1365 and https://www.ebi.ac.uk/ena/data/view/PRJEB5984).

## Abbreviations

3’RACE: 3′ rapid amplification of cDNA ends
APA: Alternative polyadenylation
CDS: Coding DNA sequence
DIG: Digoxygenin
dNTP: Desoxyribonucleotide triphosphate
ENA: European nucleotide archive
FPKM: Fragments per kilobase of exon per million fragments mapped
GPCR: G protein-coupled receptor
IsoSCM: Isoform structural change model
NA: Not available
ND: Not detected
OM: Olfactory mucosa
OSN: Olfactory sensory neuron
PAS: Polyadenylation signal
PC: Principal component
PCA: Principal component analysis
PolyA: Polyadenylation
RL-PAT: RNA ligation mediated polyadenylation test
UCSC: University of California, Santa Cruz
UTR: Untranslated region
