# Supplementary figures and images for: Alternative polyadenylation produces multiple 3’ untranslated regions of odorant receptor mRNAs in mouse olfactory sensory neurons
Source: BMC Genomics. 2019 Jul 12;20:577. doi: 10.1186/s12864-019-5927-3 (PMC6624953; doi:10.1186/s12864-019-5927-3)

# SuppFig 1

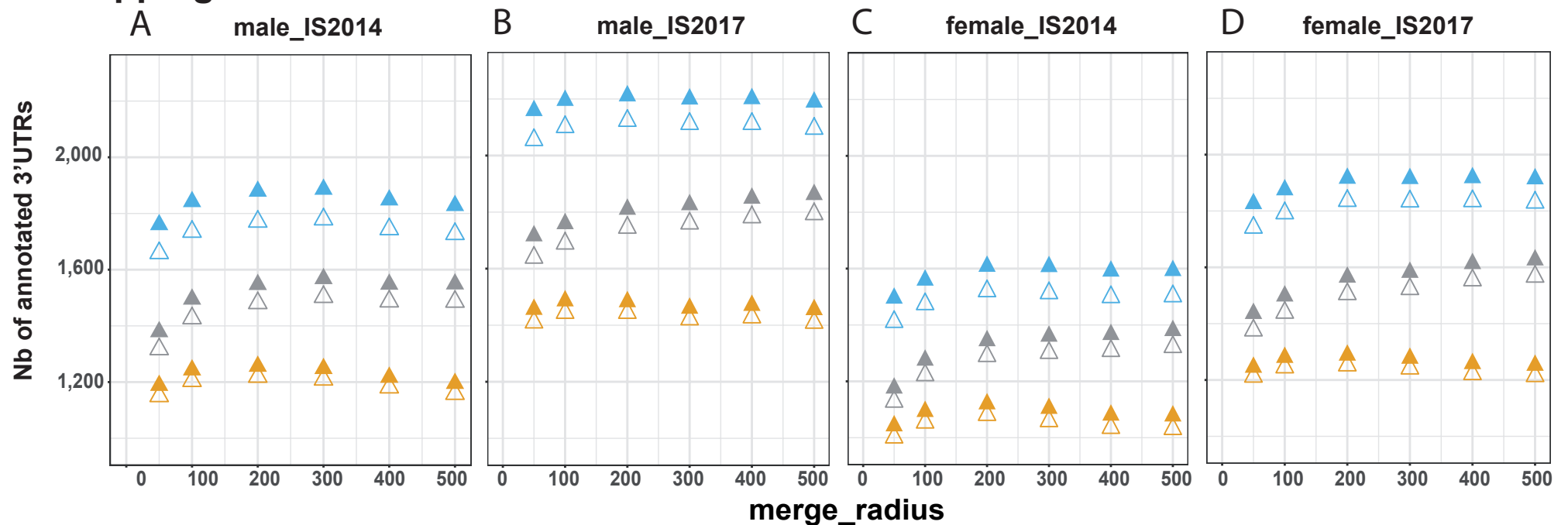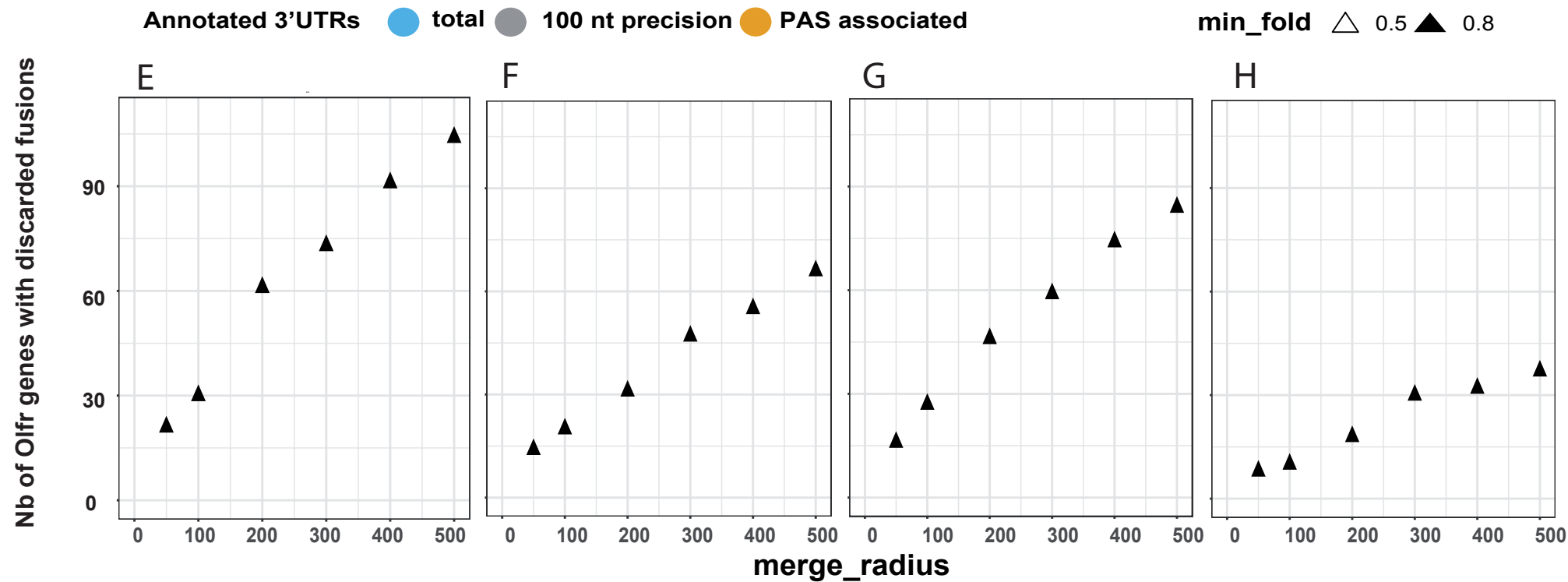

Supplement: Supplementary file 2 — Figure S1. Set up of IsoSCM min_fold and merge_radius parameters. A-D. Numbers of annotated 3’UTRs are higher when min_fold is set at 0.8 (solid triangles) as compared to default setting at 0.5 min_fold (empty triangles). Blue label = Total numbers of annotated 3’UTRs; grey label = numbers of 3’UTRs merging 3’ends under the 100-nt precision threshold; orange label = numbers of 3’UTRs matching at least one canonical AAUAAA or AUUAAA PAS in a [− 100;+ 100] window. E-F. Increasing merge_radius triggers higher numbers of Olfr genes showing annotation of chimeric exons between adjacent genes (discarded fusions). min_fold is set to 0.8 in E-F. (PDF 392 kb) [file 12864_2019_5927_MOESM2_ESM.pdf]

SuppFig 2

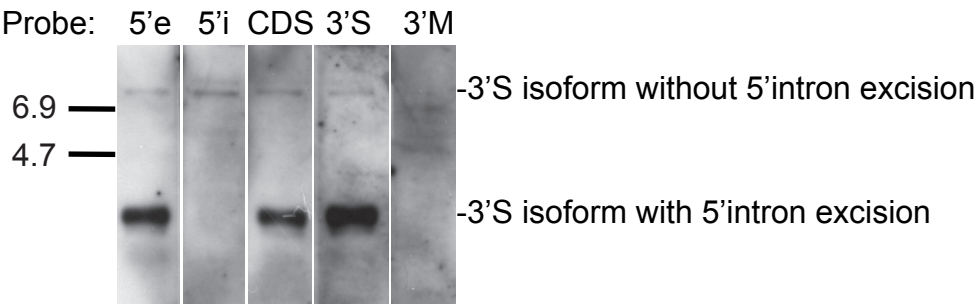

Supplement: Supplementary file 4 — Figure S2. Northern blot characterization of the intron-retaining transcript for Olfr1507. Total OM RNAs were separated on an agarose/formaldehyde gel and transferred onto a nitrocellulose membrane. The presence of Olfr1507 mRNAs was detected following hybridization with DIG-labeled antisense probes either in the 5′ exon (5’e), the 5′ intron (5’i), the CDS region (CDS), between the CDS and 3′S ends (3′S) or between the 3′S and 3’M ends (3’M) (see Table 3 for detailed probe description). The major isoform of the Olfr1507 mRNA is characterized by the absence of the 5′ intron that has been excised, and the presence of a short 3’UTR (≈3-kb dark band detected with 5’e, CDS and 3′S probes, not detected with 3’M or 5’i probes); the highest band (indicated with # in Fig. 3g) corresponds to an intron-retaining Olfr1507 mRNA bearing a short 3’UTR (> 7 kb light band detected with 5’e, 5’i, CDS and 3′S probes, not detected with 3’M probe). (PDF 1137 kb) [file 12864_2019_5927_MOESM4_ESM.pdf]

SuppFig 3

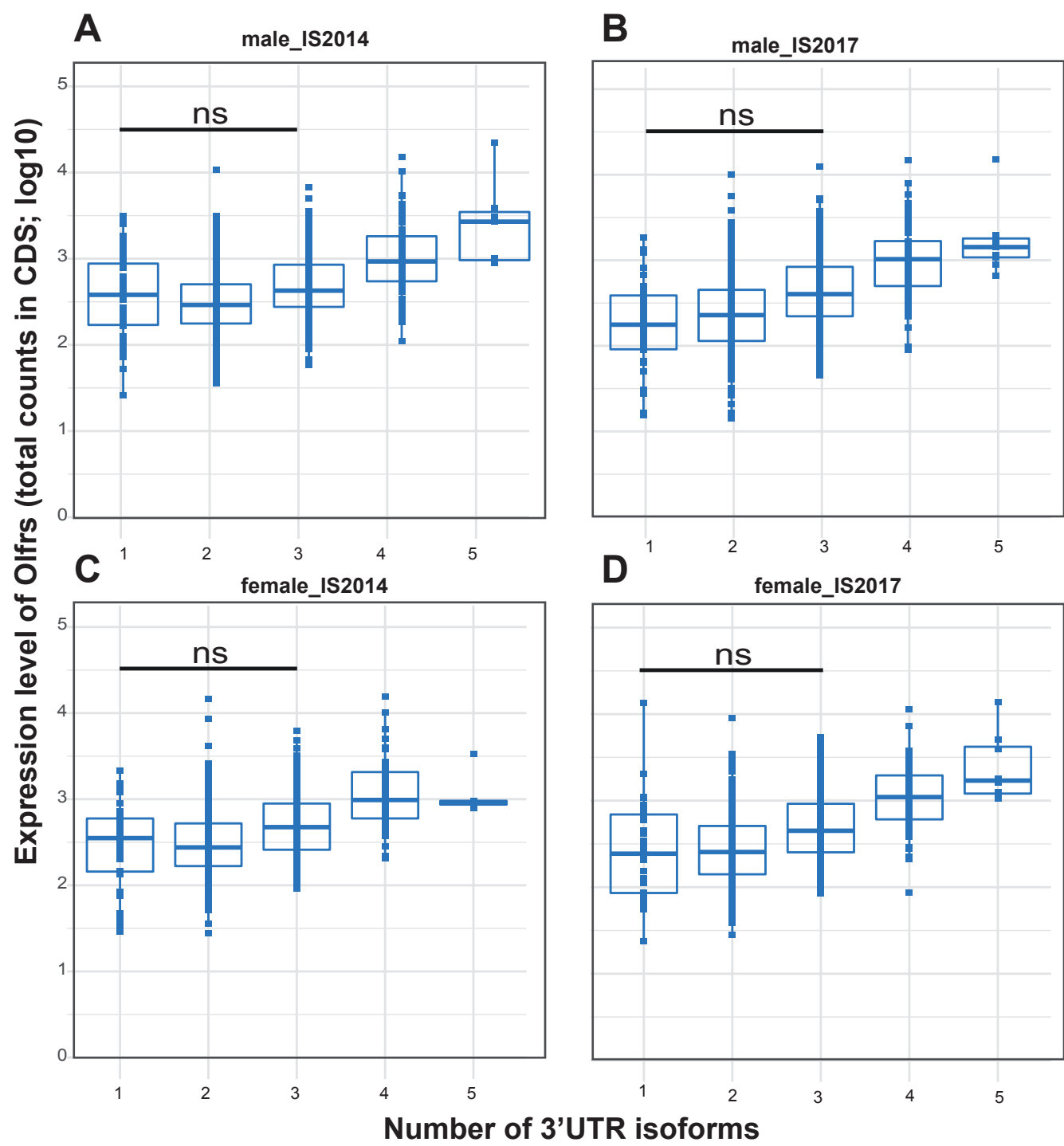

Supplement: Supplementary file 5 — Figure S3. Unbiased detection of APA for Olfr 3’UTRs in the 4 datasets. A, B, C, D. Expression levels of Olfr mRNAs (total counts in CDS) related to the number of 3’UTR isoforms per Olfr gene. One-way Kruskal-Wallis test (A: Chi square = 88.615, p < 0.0001, df = 4; B: Chi square = 144.89, df = 4, p < 0.0001; C: Chi square = 100.62, p < 0.0001, df = 4; D: Chi square = 119.06, df = 4, p < 0.0001), followed by Nemenyi test show no difference for expression levels between Olfr without APA (single 3’UTR) and Olfr with 2 or 3 3’UTR isoforms, demonstrating that, up to three 3’UTR isoforms, APA is detected independently of the expression level of the Olfr genes. However, annotation of more than 3 isoforms is biased by expression level, and we probably underestimate the number of 3’UTR isoforms (more than 3) for Olfr genes expressed at low level. (PDF 435 kb) [file 12864_2019_5927_MOESM5_ESM.pdf]

SuppFig 4

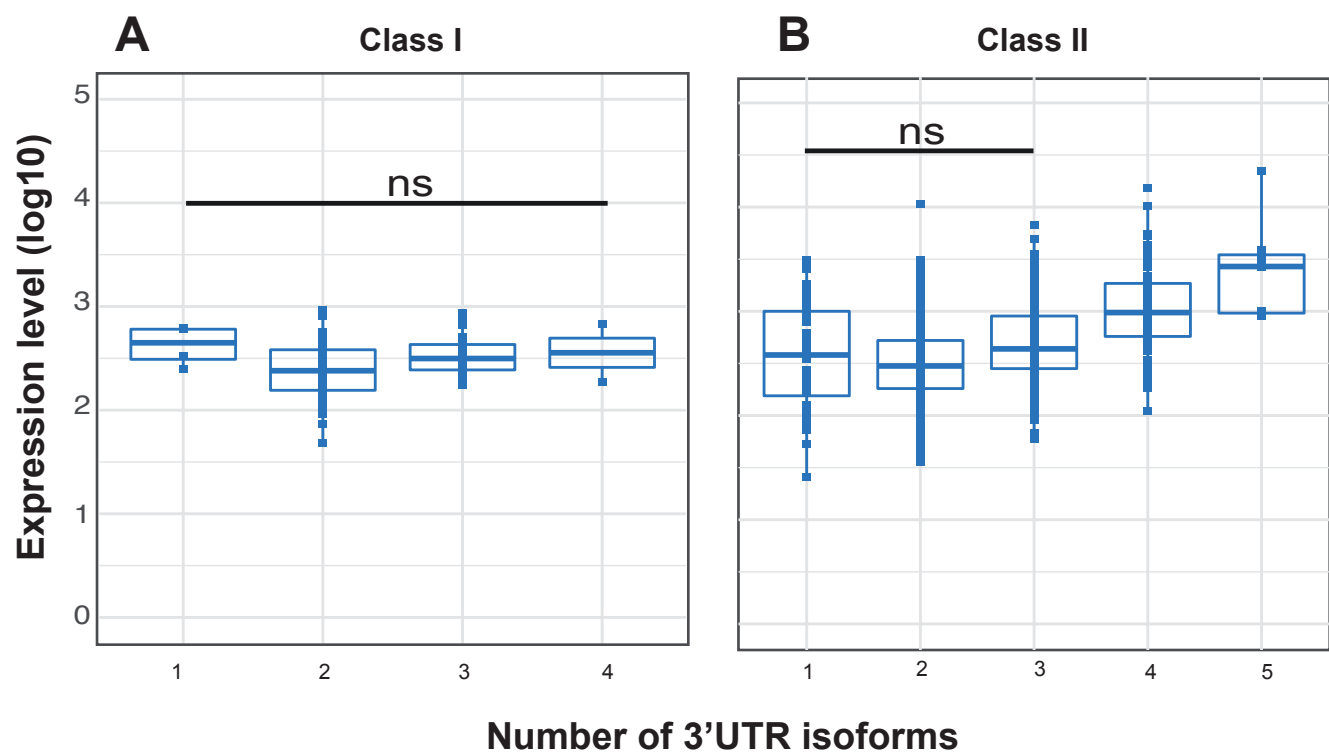

Supplement: Supplementary file 7 — Figure S4. Unbiased detection of APA for Class I or Class II Olfr 3’UTRs in the male_IS2014 dataset A. Expression levels of Class I Olfr mRNAs (total counts in CDS) related to the number of 3’UTR isoforms per Olfr gene. One-way Kruskal-Wallis test (Chi-squared = 5.5307, df = 3, p-value = 0.136) shows no difference for expression levels between Olfr without APA (single 3’UTR) and others. B. Expression levels of Class II Olfr mRNAs (total counts in CDS) related to the number of 3’UTR isoforms per Olfr gene. One-way Kruskal-Wallis test (Chi-squared = 80.875, df = 4, p < 0.0001), followed by Nemenyi test show no difference for expression levels of Class II Olfr mRNAs with up to three 3’UTR isoforms, demonstrating that APA is detected independently of the expression level of the Class II Olfr genes. Annotation of more than 3 isoforms for Class II Olfr genes is biased by expression level, and we probably underestimate the number of 3’UTR isoforms (more than 3) for low expression Class II Olfr genes. (PDF 384 kb) [file 12864_2019_5927_MOESM7_ESM.pdf]

**SuppFig 5**

**A**

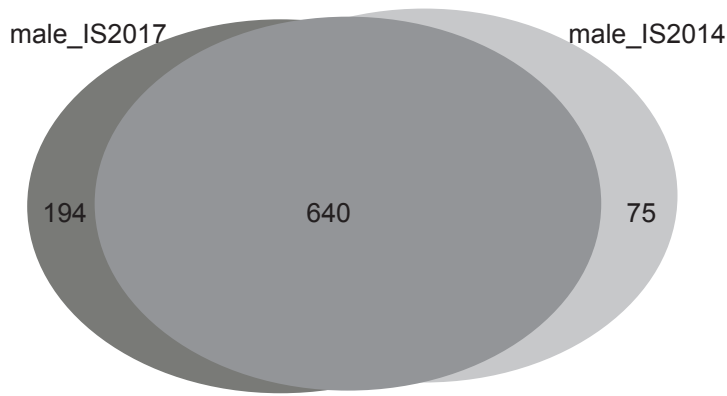

**B**

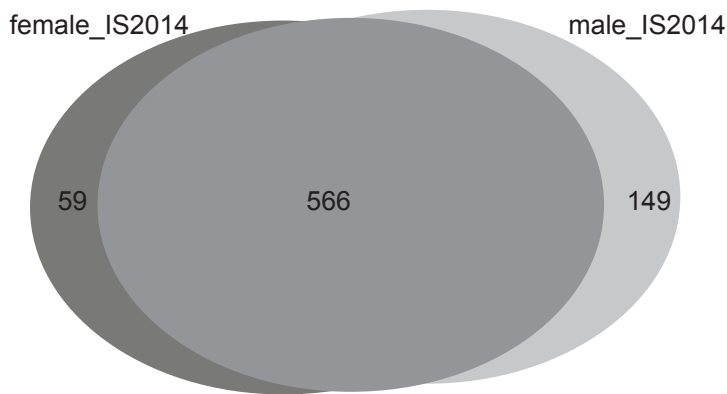

**C**

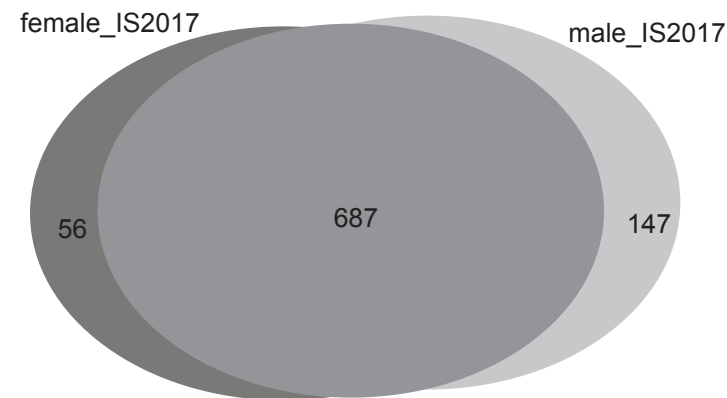

**D**

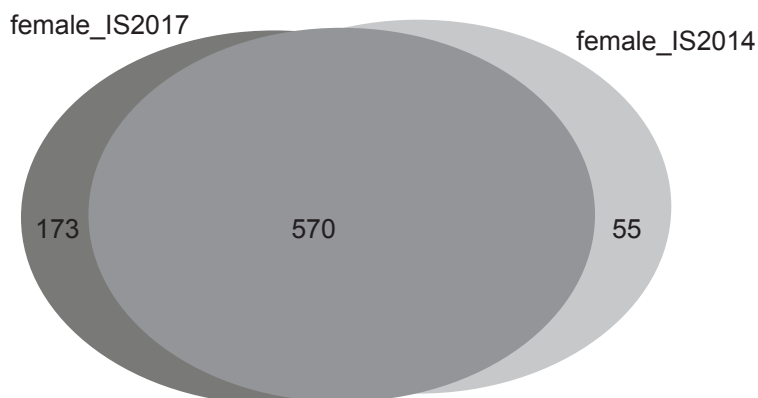

**E**

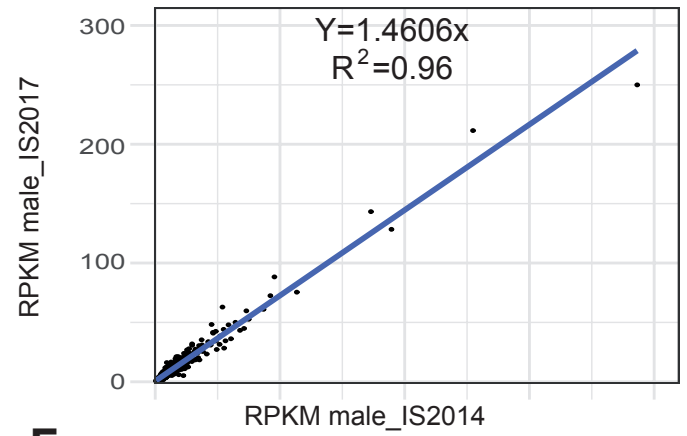

**F**

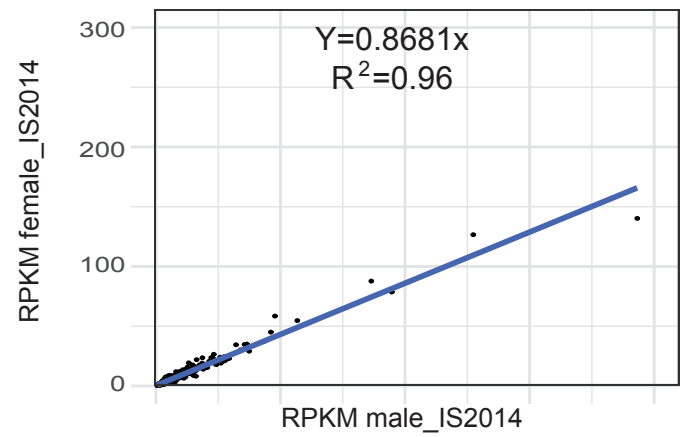

**G**

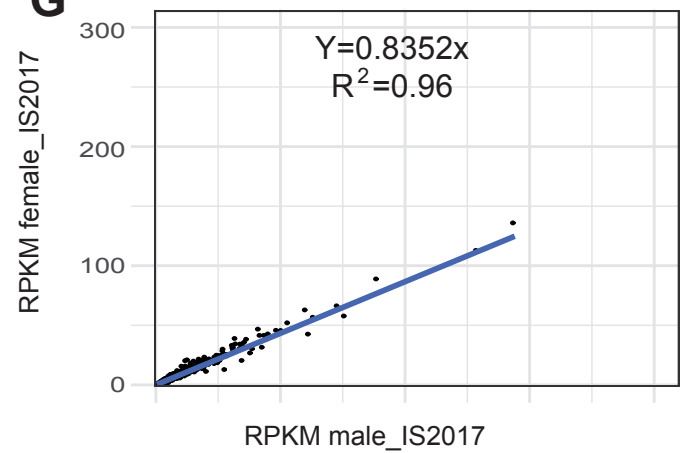

**H**

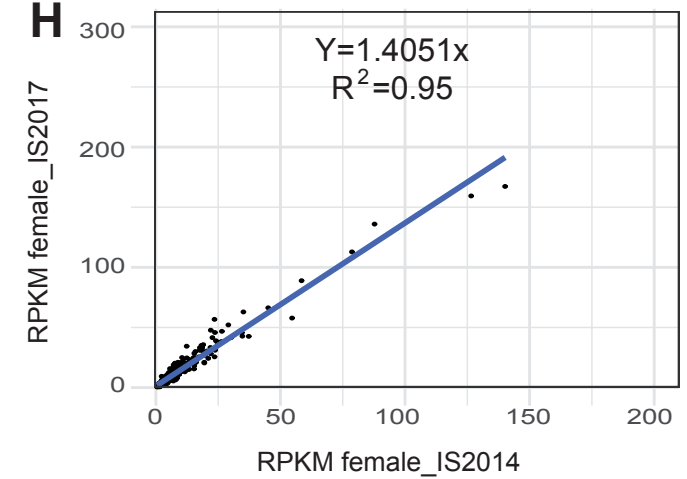

Supplement: Supplementary file 8 — Figure S5. Comparison of the subsets of annotated Olfr genes between the 4 datasets. A-D. Venn diagrams showing the numbers of Olfr genes with annotated 3’UTR(s) in 2 different datasets. The intersection of the 2 ellipses represent the common Olfr genes retrieved in the 2 datasets. E-H. Correlation of the expression levels between 2 datasets restricted to common Olfr with annotations in both datasets. The expression levels of Olfr genes are similar in the male and female datasets from each experiment (2014 or 2017) as previously shown in [26]. Pearson equation and coefficient were used to estimate the fitting level (p < 0.05) (PDF 570 kb) [file 12864_2019_5927_MOESM8_ESM.pdf]

SuppFig 6

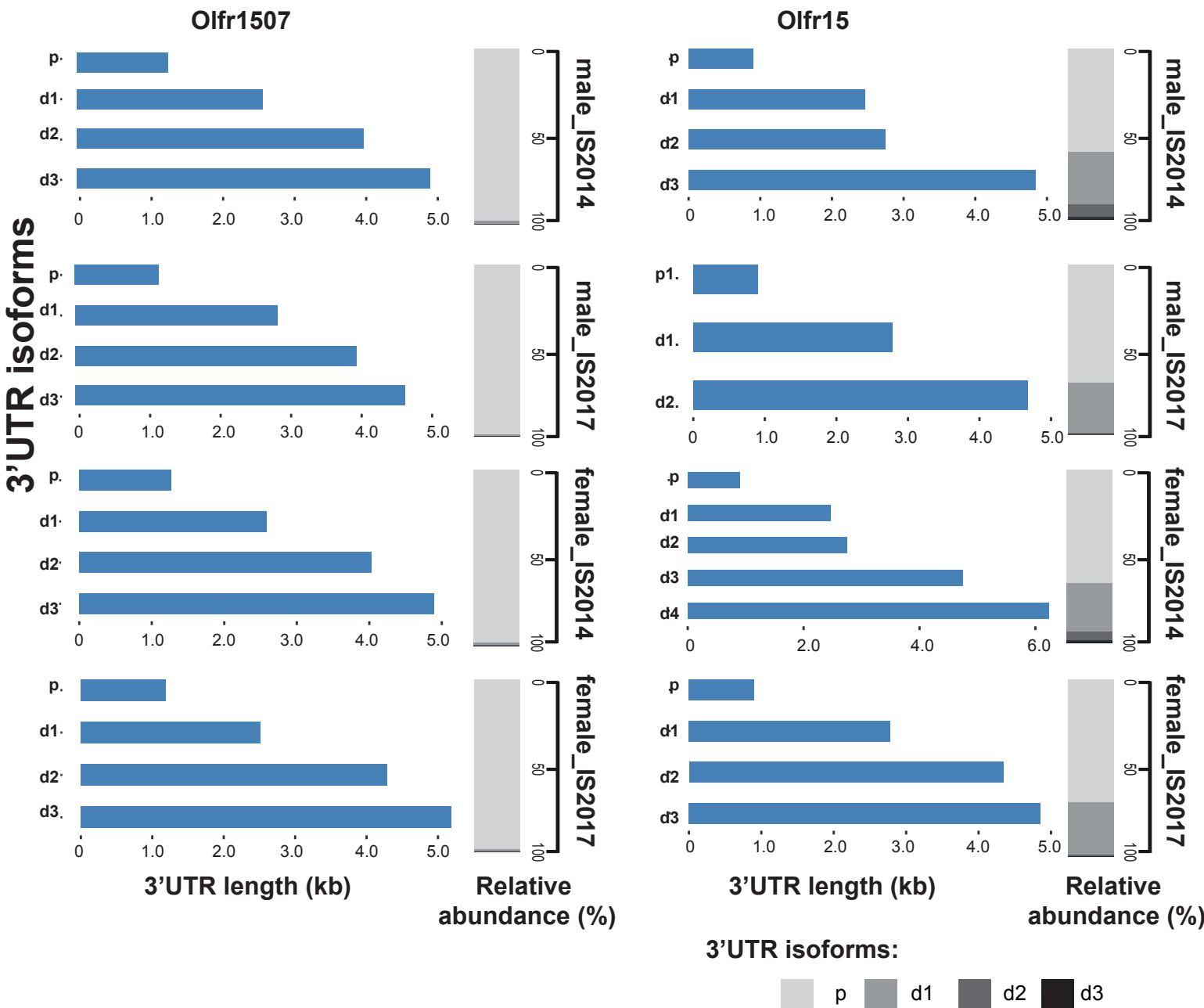

Supplement: Supplementary file 9 — Figure S6. Alternative 3’UTR isoforms identified for 2 pilot genes in the 4 datasets. Graphical representation of the alternative 3’UTR isoforms annotated for Olfr1507 or Olfr15 in terms of 3’UTR length (left panels; blue bars) and relative abundance (right panels). (PDF 393 kb) [file 12864_2019_5927_MOESM9_ESM.pdf]

SuppFig 7

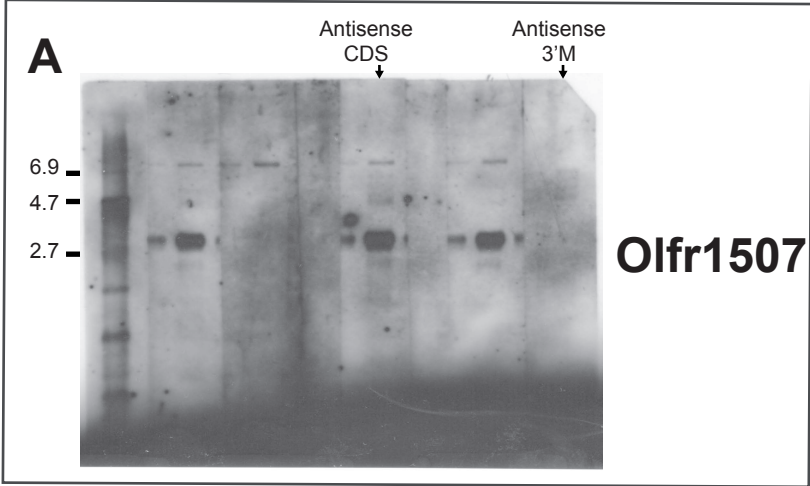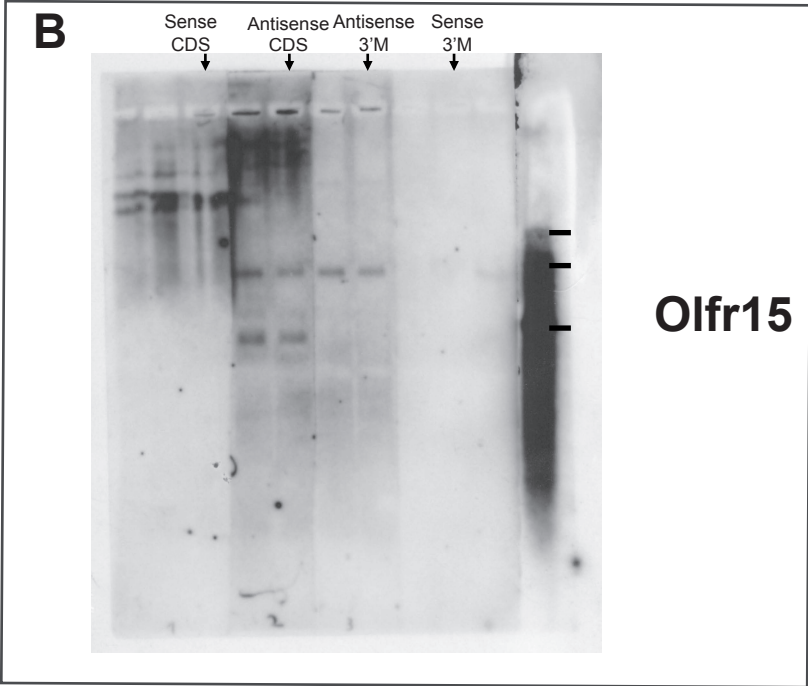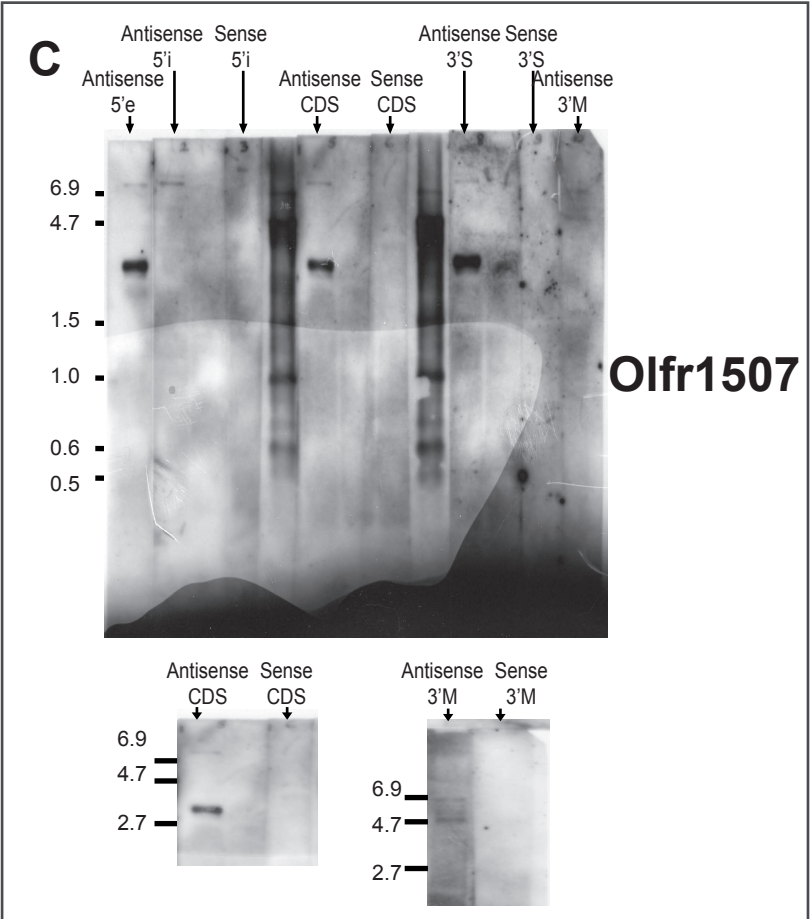

Supplement: Supplementary file 15 — Figure S7. Whole northern blots and demonstration of the probe specificity for Olfr1507 and Olfr15 northern blot experiments. Whole northern blots correspond to Fig. 3g (A) and h (B) and Additional file 4: Figure S2 (C). The specificity of the antisense probes used in northern blots was confirmed by the absence of signals with the sense probes for Olfr1507 (B-C) or Olfr15 (A). See the corresponding figure legends and Table 3 for the detailed description of the probes. (PDF 363 kb) [file 12864_2019_5927_MOESM15_ESM.pdf]
